# Carotenoid Cocktail Produced by An Antarctic Soil *Flavobacterium* with Biotechnological Potential

**DOI:** 10.3390/microorganisms9122419

**Published:** 2021-11-24

**Authors:** Paulina Pradel, Nancy Calisto, Laura Navarro, Andrés Barriga, Nicolás Vera, Carlos Aranda, Robert Simpfendorfer, Natalia Valdés, Gino Corsini, Mario Tello, Alex R. González

**Affiliations:** 1 Laboratorio de Microbiología Ambiental y Extremófilos, Departamento de Ciencias Biológicas y Biodiversidad, Universidad de los Lagos, Osorno 5290000, Chile; paulina.pradel@ulagos.cl (P.P.); nicolas.ulagos@gmail.com (N.V.); 2 Laboratorio de Bacteriología Molecular, Instituto de Ciencias Biomédicas, Facultad de Ciencias de la Salud, Universidad Autónoma de Chile, Santiago 8320000, Chile; nancy.calisto@umag.cl (N.C.); laura.navarro@cloud.uautonoma.cl (L.N.); gino.corsini@uautonoma.cl (G.C.); 3 Centro de Investigación y Monitoreo Ambiental Antártico (CIMAA), Departamento de Ingeniería Química, Facultad de Ingeniería, Universidad de Magallanes, Punta Arenas 6200000, Chile; 4 Unidad de Espectrometría de Masas-CEPEDEQ, Facultad de Ciencias Químicas y Farmacéuticas, Universidad de Chile, Sergio Livingstone 1007, Santiago 8320000, Chile; anbarr@ciq.uchile.cl; 5 Departamento de Ciencias Biológicas y Biodiversidad, Universidad de los Lagos, Osorno 5290000, Chile; caranda@ulagos.cl; 6 Departamento de Acuicultura y Recursos Agroalimentarios, Universidad de los Lagos, Osorno 5290000, Chile; robert.s@ulagos.cl; 7 Facultad de Química y Biología, Departamento de Biología, Laboratorio de Metagenómica Bacteriana, Universidad de Santiago de Chile, Santiago 8320000, Chile; natalia.valdes.parra@gmail.com (N.V.); mario.tello@usach.cl (M.T.)

**Keywords:** Antarctic *Flavobacterium*, carotenoids, lutein, zeaxanthin, aquaculture

## Abstract

Carotenoids are highly important in pigmentation, and its content in farmed crustaceans and fish correlates to their market value. These pigments also have a nutritional role in aquaculture where they are routinely added as a marine animal food supplement to ensure fish development and health. However, there is little information about carotenoids obtained from Antarctic bacteria and its use for pigmentation improvement and flesh quality in aquaculture. This study identified carotenoids produced by Antarctic soil bacteria. The pigmented strain (CN7) was isolated on modified Luria–Bertani (LB) media and incubated at 4 °C. This Gram-negative bacillus was identified by 16S rRNA analysis as *Flavobacterium segetis*. Pigment extract characterization was performed through high-performance liquid chromatography (HPLC) and identification with liquid chromatography–mass spectrometry (LC–MS). HPLC analyses revealed that this bacterium produces several pigments in the carotenoid absorption range (six peaks). LC–MS confirms the presence of one main peak corresponding to lutein or zeaxanthin (an isomer of lutein) and several other carotenoid pigments and intermediaries in a lower quantity. Therefore, we propose CN7 strain as an alternative model to produce beneficial carotenoid pigments with potential nutritional applications in aquaculture.

## 1. Introduction

The Antarctic continent is an extreme environment, not only due to its low temperatures, but also because of high UV radiation exposure [1,2]. Therefore, microorganisms have developed mechanisms to reduce the impact and damage produced by different abiotic stress factors [3]. In particular, UV exposure triggers the generation of reactive oxygen species (ROS), which could lead to oxidative stress if antioxidant cell mechanisms are overwhelmed with pro-oxidant agents [4,5]. The toxic effect induced by ROS includes oxidative damage in DNA, lipids, and proteins leading to metabolic malfunctioning [4]. The main mechanisms to reduce this damage are the synthesis of ROS scavenging enzymes and pigments [6].

Pigments are present in several microorganisms and they are described as an integral part of the complex membrane structure, and influence membrane fluidity by increasing its rigidity and mechanical strength [7,8]. Carotenoid pigments represent the largest and most diverse class of natural products known to mankind [9], varying in color from yellow to orange–red depending on their chemical structure (terpenoids), and hence are responsible for the colors seen in different microorganisms, plants, and animals living especially under extreme conditions [10,11].

Natural pigments like carotenoids have high antioxidant effects through their photoprotective action [3]. Furthermore, several epidemiological studies strongly suggest that consumption of carotenoid-rich foods reduces the incidence of diseases such as cancer, cardiovascular disease, cataracts, diseases related to low immune function, and other degenerative diseases [12,13,14].

In vertebrates, body coloration plays an important role in camouflage, communication, ecological interactions, and speciation [15]. In fish, carotenoid-based body coloration is influenced by diet and body condition and is positively correlated to mating success and social dominance [16]. Arous et al. (2014) also described the carotenoids’ involvement in fish health, growth performance, and survival [17].

Carotenoid supplementation in farmed salmon and red sea bream was demonstrated to increase ovary development, fertilization, hatching, and larval growth [18]. Currently, this pigment type is used in the aquaculture industry, mainly in salmon food, for the nutritional properties mentioned above and because the color of fish is a quality criterion used by consumers to assess the nutritive value, health, freshness, and taste of salmonids [12,19]. Consequently, natural carotenoid sources show an increasing demand in the food industry.

This study thus aimed to identify all the carotenoids produced from psychrotrophic CN7 bacterium and reflect on its use as a potential source of carotenoids for aquaculture applications.

## 2. Materials and Methods

### 2.1. Bacterial Sample and Morphological Characterization 

A soil sample was collected from Antarctic soil on King George Island (S62°10′42.5″, W58°55′59.5″). An orange pigmented colony (named as CN7) was isolated and grown on modified LB agar (3.3 g/L NaCl, 1.7 g yeast extract, 3.3 g/L peptone, 15 g/L agar-agar), and incubated aerobically at 4 °C. Morphological characterization was performed using Gram staining [20] and microscopy analysis was carried out using an optical light microscope (Olympus CX21, Tokyo, Japan) with 100× of magnification.

### 2.2. DNA Extraction and Amplification of the 16S rRNA Gene of CN7 Strain

DNA extraction of the CN7 strain was performed using the phenol: chloroform (1:1) method after cell lysis mediated by lysozyme (5 µg/mL) and 10% SDS. DNA precipitation was achieved using absolute ethanol and was stored overnight at −20 °C. Its quality was assessed by determining the 260/280 nm ratio [21]. 

Subsequently, amplification of 16S rRNA gene of CN7 strain was performed with the following PCR program: denaturation at 94 °C for 5 min followed by 35 cycles at 94 °C for 30 s, primer binding at 55 °C for 30 s, and extension at 72 °C for 1 min, with a final extension at 72 °C for 10 min. The primers used for the amplification of the 16S gene were 27F (5′-AGA GTT TGA TCM TGG CTC AG-3′) [22] and 1492R (5′-TAC GGY TAC CTT GTT ACG ACT T-3′) [23]. The PCR product (1420 pb) was separated on a 1.5% agarose gel in 1x TAE buffer, stained with ethidium bromide, and visualized with UV light using a transilluminator. PCR products were gel purified, sequenced and sent to OMICS Inc. The identity of the strain was determined through a bioinformatic analysis.

The phylogenetic tree was then constructed with the sequences obtained using ClustalW2 and MEGA X. The nucleotide sequence was deposited in the NCBI GenBank database (https://www.ncbi.nlm.nih.gov/ (accessed on 6 June 2014) under accession number (KJ943556.1).

For the phylogenetic analysis, an initial search for homologous sequences of the 16S ribosomal RNA of CN7 strain (KJ943556.1) was performed using the BLAST software Version 2.7.1 and the Blastn search application [24]. The search was performed using the uncured database of 16S rRNA sequences (bacteria and archaea) from the National Center for Biotechnology Information (NCBI). The matches were ranked by E-value and filtered by coverage (>95%). The 30 hits with the lowest E-value were chosen for further phylogenetic analysis, including as an outer group the 16S rRNA of *Cytophaga hutchinsonii* ATCC 33406 (NR 102866.1). All sequences were aligned with the ClustalW2 software [25] and the phylogenetic tree was built with the neighbor-joining method using the Mega X program [26]. The analysis was performed using 10,000 repetitions bootstrap and the Jukes–Cantor distance method. All ambiguous positions were removed for each sequence pair (pairwise removal option).

### 2.3. CN7 Pigment Extraction

The CN7 strain was grown aerobically in a modified LB liquid medium at 20 °C. Cells were centrifuged and the supernatant discarded. In order to extract the pigment, the sample was sonicated (Q55 Sonicator, Sonica) three times for 5 s using acetone to improve pigment extraction. The latter was removed from the combined acetone extract by rotary evaporation at 40 °C and stored in darkness at −20 °C. Finally, the pigments were dried using nitrogen gas.

### 2.4. High-Performance Liquid Chromatography (HPLC) and UV-Vis Detection

The high-pressure liquid chromatography analysis of isolated carotenoids was performed under gradient conditions with a JASCO HPLC, PU-2089 Plus pump, reverse phase Kromasil RP-C18 column (3.5 μm particle size, 15 cm × 4.6 mm ID) using a diode array detector (MD-2015 Plus) set to a wavelength (λdet) range between 220 and 700 nm and a flow rate of 1 mL/min, at 22 °C. The injection volume was 20 μL. The gradient conditions for the mobile phase water/methanol (W/M) were as follows: the initial mobile phase at 15% W and 85% M was linearly changed to 100% M over 21 min. To identify the typical UV/visible spectrum of carotenoids, we injected in the HPLC astaxanthin (0.85 µg) and canthaxanthin (4 µg) as control. Both reactive were obtained from Sigma Aldrich (St. Louis, MO, USA).

### 2.5. Liquid Chromatography–UltraViolet-Mass Spectrometry (LC–UV-MS/MS) Analysis for Pigment Identification

Pigment extracts were resuspended in methanol and examined in a LC–MS system consisting of the HPLC HP1100 (Agilent Technologies Inc., CA-USA) coupled to the electrospray-ion trap mass spectrometer Esquire 4000 (Bruker Daltonik GmbH, Bremen, Germany). For chromatographic separation, a C18 column (300 × 4.0 mm, 5 μm and 120 Å, Supelcosil LC-18, Supelco Inc., Bellefonte, PA, USA) was used. The column outlet was connected to a split that divided the flow to the UV-vis detector and to the mass spectrometer. The separation of 20 μL of extracts was performed at room temperature using the following program: 0.0–37.5 min 88–85% B, 37.5–39.0 min 85-88% B and 39.0–45.0 min 88% B using 10 mM ammonium formate in water as phase A and acetonitrile as phase B at a flow of 1.0 mL/min [27]. The electrospray ionization process was carried out using nitrogen at a nebulization temperature of 350 °C, a nebulization pressure of 25 psi, and a nebulizer gas flow of 6.5 L/min. Mass spectra were acquired in positive polarity and UV-vis detection was acquired at a wavelength of 450 nm. The MS^2^ acquisitions were obtained in the auto-MS/MS mode considering the following parameters: SmartFrag, 30–200%; fragmentation amplitude, 1.0 V; fragmentation time, 40 ms; isolation width MS/MS, 4 *m*/*z*; fragmentation enhancement, on. Visualization and analysis of the chromatograms and mass spectra was performed using DataAnalysis 3.2 (Bruker Daltonik GmbH, Bremen, Germany). The carotenoids were identified using the MassBank database (http://www.massbank.jp (accessed on 5 December 2013)) [28].

## 3. Results

### 3.1. Characteristics of the Isolated CN7 Strain

**Figure 1:**
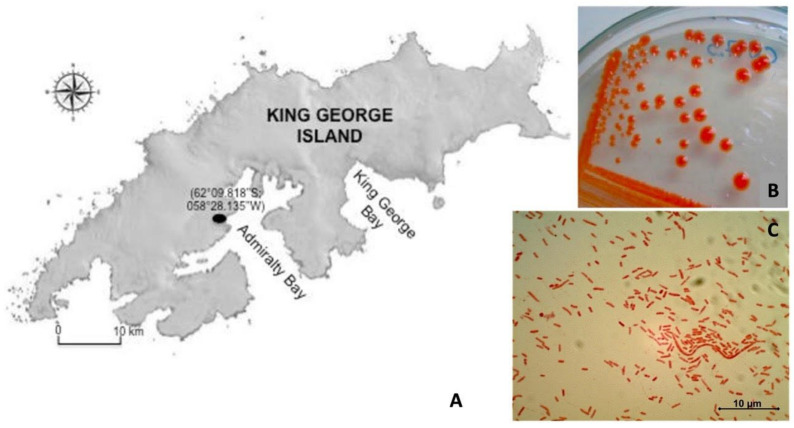
(**A**) Sample area at King George Island, Antarctica (S62°10′42.5″, W58°55′59.5″). (**B**) Intense orange colonies recovered from topsoil growth on LB modified medium. (**C**) Microscope analysis of CN7 strain. According to the Gram staining, the CN7 strain is a Gram-negative bacillus.

Antarctic pigmented bacterium (CN7 strain) was isolated from Antarctic topsoil (King George Island) (Figure 1A). The isolate obtained formed a circular colony with an intense orange color, small size with raised elevation and regular margin (Figure 1B). Gram staining revealed this strain is a Gram-negative bacillus (Figure 1C). 

### 3.2. CN7 Strain Identification

**Figure 2:**
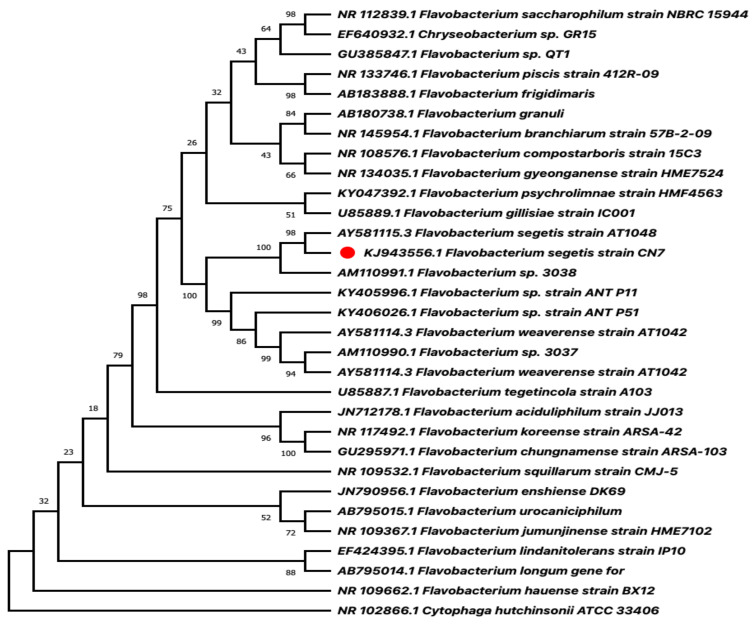
Dendrogram of phylogenetic reconstruction based on analyzing ribosomal RNA 16S of *F. segetis* strain CN7 (KJ943556.1) (red circle). Phylogenetic reconstruction was performed using the neighbor-joining (NJ) method within the Mega X program. The evolutionary distances were computed using Jukes–Cantor with the pairwise deletion option. The percentage of bootstrap values is shown next to the branches based on 10.000 bootstrap replications. The sequence of interest is shown with a red circle.

For molecular identification and strain validation, 16S ribosomal RNA sequence analysis of the CN7 strain (KJ943556.1) was performed using phylogeny (Figure 2). The rooted tree shows that the 16S ribosomal RNA sequence of the strain is grouped with the homologous sequences of microorganisms belonging to the genus *Flavobacterium*. Specifically, the isolate of interest has a 99.3% of identity with *Flavobacterium segetis* AT1048. Considering that the threshold identity to be assigned to the same species is 97% [29], this result allows it to classify the isolated as members of *F. segetis* species. Further characterization and comparison using the entire sequence of *F. segetis* AT1048 genome will prove or refute this taxonomic classification.

### 3.3. Carotenoids Fractionation from CN7 Strain

**Figure 3:**
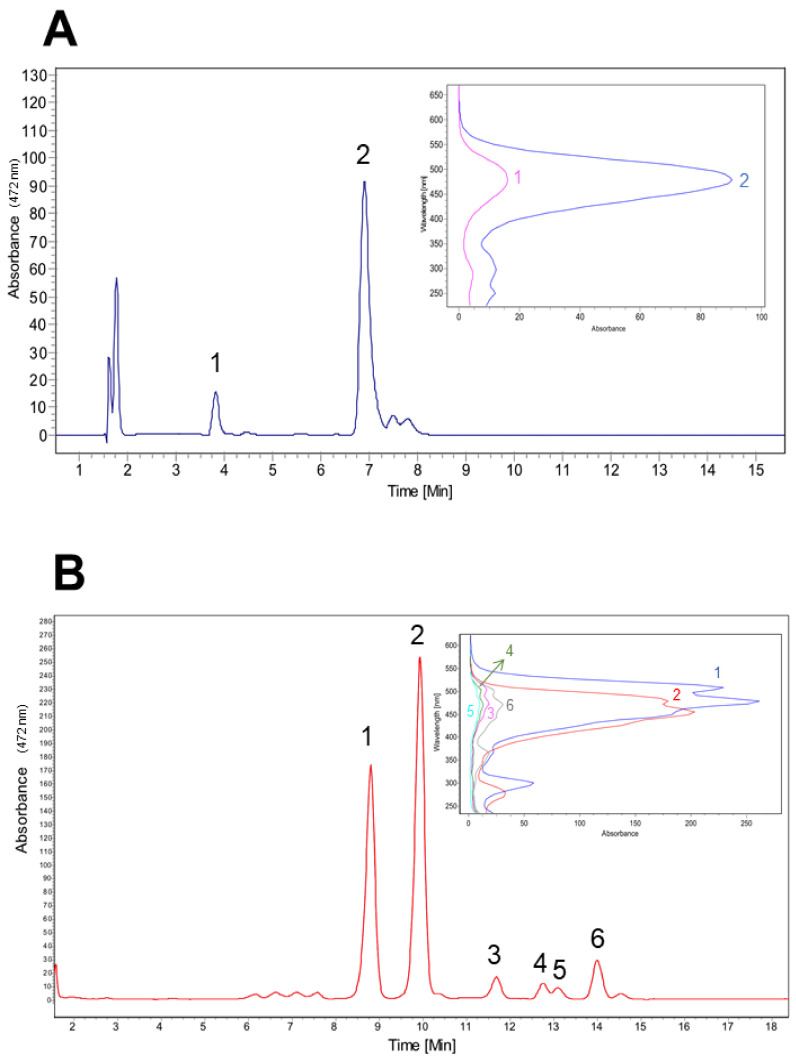
HPLC profile of total pigments extracted from isolated CN7. (**A**) Commercial astaxanthin (peak 1) and canthaxanthin (peak 2); insert shows both UV-visible spectrograms. (**B**) Chromatogram (recorded at 472 nm) of acetone-extracted carotenoids, showing two main peaks (1 and 2) with larger absorbance levels; insert shows UV-visible spectrograms of the main 6 carotenoid peaks extracted from CN7 strain.

Preliminary HPLC-DAD analysis of the pigments present in CN7 isolate shows the maximum absorption peaks were present in the visible range (450–500 nm) and correspond to reddish xanthophyll carotenoid compounds (Figure 3B) [30]. Six mean carotenoid peaks were obtained from the biomass of *F. segetis* and were chromatographically separated. The six peaks observed in Figure 3B had the following retention times and maximum absorption wavelengths for each peak: peak 6 = 13.99 min (λmax = 470 nm), peak 5 = 13.08 min (λmax = 476 nm), peak 4 = 12.79 min (λmax = 464 nm), peak 3 = 11.68 min (λmax = 475 nm), peak 2 = 9.95 min (λmax = 475 nm), and peak 1 = 8.8 min (λmax = 450 nm). We used astaxanthin and canthaxanthin (from Sigma Aldrich fine chemicals) as commercial standards, with retention times of 3.85 and 6.90 min (λmax = 478 nm both; insert in Figure 3A). The HPLC data corresponds to the total amount of carotenoids extracted by acetone. This analysis showed the main CN7 strain xanthophyll has maximum absorption at 475 nm and a shoulder at 515 nm (Figure 3B). To characterize and identify each peak obtained in the HPLC-DAD study, we submitted the pigment cocktail to LC–MS analysis under the previously mentioned conditions.

### 3.4. Carotenoids Identification from Antarctic Isolated CN7

**Figure 4:**
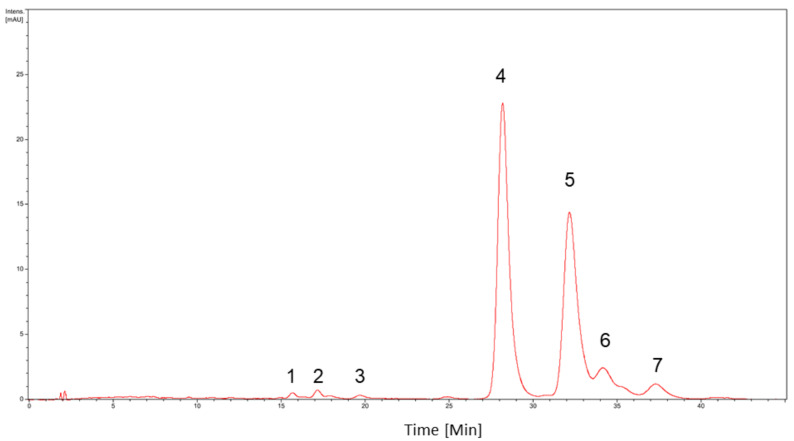
LC-UV-MS/MS profile at 450 nm of carotenoid pigments produced by strain CN7. Identification analysis was carried out using the mass spectra determined for each peak. The proposed identifications are detailed in Table 1.

Figure 4 shows the chromatogram obtained from LC-UV/vis-MS/MS analysis. For each of the chromatographic peaks observed, their corresponding mass spectra were obtained, annotating the *m*/*z* signals with their corresponding fragmentations (Table 1). For the pigments identification, we considered: (i) the determination of the main *m*/*z* signal of the chromatographic peak based on adduct formation and probable in-source fragmentation, (ii) a comparison of experimental fragmentation with that described in the literature for carotenoids and (iii) a comparison of experimental fragmentation versus fragmentation available in the MassBank database (http://www.massbank.jp (accessed on 5 December 2013)) [28].

Peak 1 (RT 15.7 min) (Figure S1) presented the signal *m*/*z* 585 in its mass spectrum which, based on its fragmentation, would correspond to capsanthin (a keto-carotenoid), lutein-5,6-epoxide (an epoxy-carotenoid) or caloxanthine (an hydroxycarotenoid). One difficulty in identifying carotenoids is the limited availability of fragmentation spectra as well as the presence of an abundant number of structural isomers. The fragmentation analysis of *m*/*z* 585 would suggest the presence of three hydroxyl groups given the observation of *m*/*z* 566 ([M+H-H_2_O]^+^), *m*/*z* 549 ([M+H-2H_2_O]^+^) and *m*/*z* 531 ([M+H-3H_2_O]^+^) (Figure S2), which would eliminate capsanthin and lutein-5,6-epoxide since they contain 2 hydroxyls while caloxanthin (=hydroxyxanthin) contains 3 hydroxyls (Figure S3). On the other hand, capsanthin and lutein-5,6-epoxide would not be produced by microorganisms unlike caloxanthin that was described for bacteria of the genus Erythrobacter and for different genera of cyanobacteria (e.g., Calothrix, Synechococcus, Anacystis) based on the information available in Carotenoids Database (http://carotenoiddb.jp (accessed on 22 April 2021)) and BioCyc Database Collection (https://biocyc.org/ (accessed on 22 April 2021).

In peak 2 (RT 17.2 min) (Figure S4), the signal *m*/*z* 584 was observed, which according to its fragmentation would correspond to capsanthin, lutein-5,6-epoxide or caloxanthin. The examination of fragmentation showed a high intensity signal *m*/*z* 564 and a low intensity signal *m*/*z* 551 which would correspond to the loss of one and two molecules of water, respectively, suggesting the presence of two hydroxyl groups; the presence of a third hydroxyl group was not clear (Figure S5). Based on the antecedents mentioned above, the observed compound would correspond to a caloxanthin isomer (Figure S6). The signal *m*/*z* 413 was also observed; however, it was not identified (Figure S7).

Peak 3 (RT 19.7 min) (Figure S8) presented the signals *m*/*z* 284 and *m*/*z* 567 that would correspond to the protonated and protonated dimer forms of a compound of mass 283.6 g/mol. Both the mass of the compound and its fragmentation are very similar to those described for the apocarotenoid compound 15-apo-carotenal [36] (Figures S9 and S10).

Peak 4 (RT 28.2 min) (Figure S11) presented the signal *m*/*z* 568 which, based on its fragmentation, would be identified as lutein or zeaxanthin (both hydroxycarotenoids). From the spectrometric point of view, the discrimination between both compounds is complex because they are structural isomers. However, some experimental observations by different authors indicate that lutein tends to exhibit a fragment *m*/*z* 551 of high intensity [27,31,38] as well as the presence of characteristic fragments such as *m*/*z* 430 or *m*/*z* 495 [35]. The fragmentation spectrum showed a low intensity signal *m*/*z* 551 (4.9%) and an absence of *m*/*z* 495, thus suggesting that the compound most likely corresponded to zeaxanthin (Figures S12 and S13). However, it must be considered that the differences in the fragmentation energies used are not similar and could alter the observation of fragments. Peak 4 from Figure 4 would correspond to peak 1 of Figure 3B, based on the absorption spectrum a %III/II value of 20 was determined, which is ranged between the reported values for zeaxanthin that reinforces the proposed identification [32,40].

Peak 5 (RT 32.1 min) (Figure S14) presented the signal *m*/*z* 621 whose MS/MS spectrum showed the fragments *m*/*z* 603 ([M-H_2_O]^•+^), *m*/*z* 589 ([M-CH_3_OH]^•+^), *m*/*z* 529 ([M-92]^•+^), *m*/*z* 515 ([M-106]^•+^) and *m*/*z* 483 ([M-CH_3_OH-106]^•+^) (Figure S15). The loss of 92 and 106 Da corresponds to the typical fragmentation of polyene chains of carotenoids. The loss of water suggests the presence of hydroxylations and the elimination of methanol would indicate the presence of methoxy groups. We observed the coelution of *m*/*z* 598 (Figure S14) would correspond based on its fragmentation to 3,4-dihydrospyriloxanthine (a hydroxycarotenoid) (Figures S16 and S17) that was previously described as produced by *Rhodospirillum rubrum*. Peak 5 (Figure 4) would correspond to peak 2 of the chromatogram of Figure 3B, the absorption spectrum presented a profile similar to peak 1 but shifted at higher wavelengths, and this suggests a longer chain length or an acyclic structure. Information consistent with that observed by mass spectrometry.

Peak 6 (RT 34.2 min) (Figure S18) corresponded to the mixture of an isomer of 3,4-dihydrospirilloxanthin (Figures S19 and S20) and lutein/zeaxanthin (Figures S21 and S22). The examination of the fragments used for differentiation between lutein and zeaxanthin showed a signal *m*/*z* 551 of high intensity (53%) apart from the presence of *m*/*z* 494 suggesting that it would correspond to lutein. Peak 6 (Figure 4) would correspond to peak 3 of Figure 3B, based on the absorption spectrum a %III/II value of 54 was determined, which is ranged between the reported values for lutein [32,40,42]. Absolute confirmation should be considered compound purification. 

For peak 7 (RT 37.3 min) (Figure S23) the signal *m*/*z* 598 was identified as an isomer of 3,4-dihydrospyrilloxanthin (Figures S24 and S25). The observed *m*/*z* 568 presented fragmentation that would indicate the presence of hydroxyechinenone (a keto-carotenoid) (Figures S26 and S27). The identification of *m*/*z* 457 could not be determined. Peak 7 (Figure 4) would correspond to peak 6 of the chromatogram of Figure 3B, the absorption spectrum showed a lower intensity of the bands suggesting some modification in the rings that could be correlated with the presence of a keto group in the proposed hydroxyechinenone or in the unidentified signal *m*/*z* 457 (Figure S28).

## 4. Discussion

The use of natural pigments, such carotenoids, has increased over the years specially because certain synthetic food colorants have deleterious effects on human health [43]. Thus, microorganisms able to produce different kind of carotenoids have great biotechnological potential. The harsh environmental conditions present in the polar regions are natural laboratories which contribute to the improvement and maximization of the biological capacities of microorganisms for extreme environments. Carotenoids are a wide family of hydrophobic molecules that play a pivotal role to improve microbial fitness to these harsh conditions. These molecules are integral parts of the cytoplasmic membranes present in bacteria, archaea, and yeast. Carotenoids reinforce mechanical strength and modulate flexibility of cell membranes, reducing susceptibility to lipid peroxidation and maintaining cell integrity under extreme conditions [44,45]. 

In this article, we characterized the diversity of carotenoids produced by a single bacterial isolate from Antarctic soil identified with the name of CN7. This isolate was capable of producing up to 6 mg/L of orange pigments, of which nearly 50% correspond to lutein-like carotenoid (Figure 4). This implies that our isolate produces three times the amount reported by other *Flavobacterium* sp. at the same temperature 20 °C [46] and is the second largest carotenoids producer reported, after *Flavobacterium multivorum* ATCC 55238 which produces around 10 mg/L of carotenoids in culture. However, the carotenoids production by *Flavobacterium multivorum* ATCC 55238 is achieved at 30 °C, implying that fermentation of our isolate could require lower energy, reducing the production cost compared to the use of *F. multivorum* ATCC 55238 [47]. Future research related to determine the optimal growth temperature for carotenoid production of our isolated are necessary properly evaluate its biotechnological application.

The identification of our strain by 16S ribosomal RNA sequence and phylogeny reconstruction (Figure 2) indicate that our strain belonged to the genus *Flavobacterium*. In particular, the CN7 isolate has 99.3% identity with *F. segetis* AT1048, an Antarctic isolate belonging to a genus with reported carotenoid production [45,48]. However, this percentage of identity indicates that CN7 is a strain different from *F. segetis* species, although further characterization comparing both genomes is necessary to confirm this taxonomic classification [29].

The initial HPLC analysis allowed us to identify the pigment produced by CN7 as a cocktail of carotenoids given that it shows maximum absorption peaks in the visible range (450–500 nm) (Figure 3). Carotenoids are a family of structurally related linear polyunsaturated molecules including several isomers with different chemical and physical properties [49]. Our characterization by LC–MS of these carotenoids shows that the main carotenoids produced by CN7 belong to the lutein-like carotenoids that include its isomers zeaxanthin both comprising from 95% to 99% of the total carotenoids [50,51] (Table 1). 

The exact proportion of zeaxanthin and lutein produced is not easy to resolve given that both compounds are structural isomers, whose differences cannot be resolved using only MS [52,53]. However, previous studies have reported that the main carotenoid pigment produced by the *Flavobacterium* genus is zeaxanthin, which also correlates with our work [54,55,56,57].

Since peaks 4 and 6 contain molecules with the same mass (*m*/*z* = 568) identified as lutein or zeaxanthin, we concluded that CN7 could produce both compounds, being the main zeaxanthin under this condition of culture. Whether this proportion can change in CN7 is currently unknown but is highly plausible given that in another microorganism the main carotenoids produced depend on fermentation conditions such as temperature, media composition, stirring rate, and aeration, among others [58]. 

**Figure 5:**
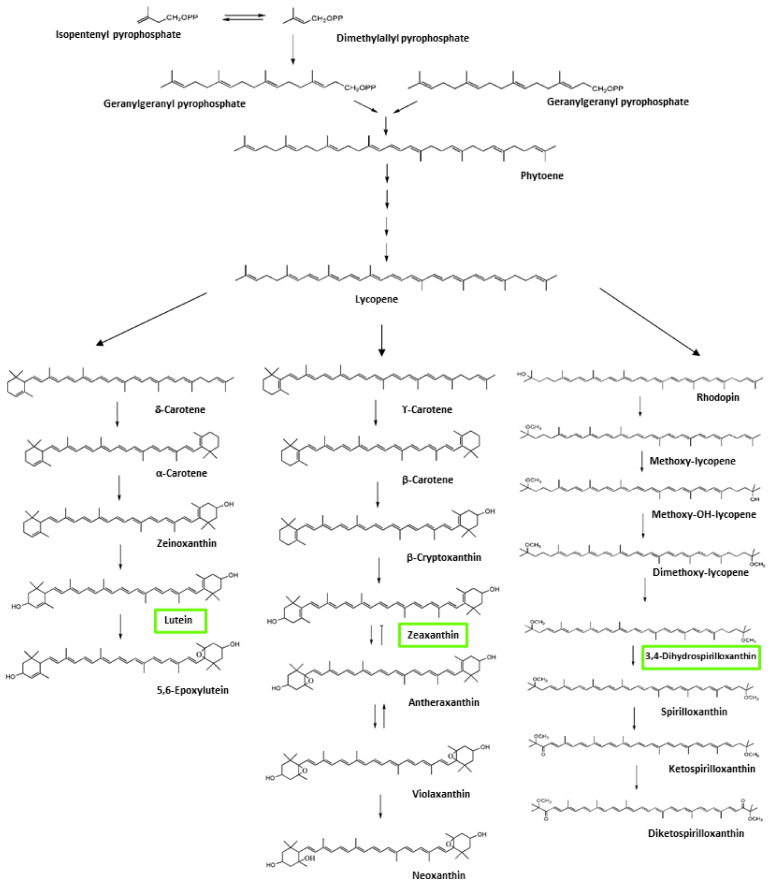
Possible metabolic pathways of CN7 for producing carotenoid pigments. Compounds determined by LC-UV/vis-MS/MS are marked in green boxes. Figure adapted from the work carried out by Kopsell and Kopsell (2006) and Chi et al. (2015) [60,61].

The presence in peak 7 of a molecule that, according to its mass, corresponds to 3,4-dihydrospirilloxanthin, suggests that CN7 contains at least two different metabolic routes for pigment production involving lycopene to produce at least two identified carotenoids or the intermediaries lutein/zeaxanthin and 3,4-dihydrospirilloxanthin. This is supported by the identification of some of its synthesis intermediaries (Figure 5). To our knowledge, there are no other reported bacteria that produce three different kinds of carotenoids. Future perspectives to probe this hypothesis could involve full genome sequencing and the use of bioinformatics tools to determine all the pigment production biochemical pathways present in this microorganism [59].

Further experiments are needed to establish the exact identity of the compounds, since there are limitations in the identification of carotenoids due to the scarcity of the fragmentation spectrum, in addition to a large number of isomers. The use of electrospray ionization (ESI) as a source of ionization also restricts the identification of other carotenoids because this source is used mainly for compounds with ionizable polar groups, so new studies should use atmospheric pressure chemical ionization (APCI) that would allow the identification of polar and apolar carotenoids. Additional studies should include purification, characterization by high-resolution mass spectrometry and NMR.

As the LC–MS shows, CN7 would produce smaller amounts of other pigments such as caloxanthin, 3,4-dihydrospirilloxanthin, hydroxyechinenone, and 15-apo-carotenal. The presence of minority pigments must be taken with caution since the extraction, handling and storage processes can alter their presence. For example, it is reported that the extraction temperature or exposure to light can favor isomerization, in this case, mass spectrometry would not tell us much about it, but the absorbance spectra in our case showed a peak at low wavelengths that would indicate the presence of cis forms [62]. The cis form is the most prone to oxidation processes such as the formation of epoxycarotenoids or apocarotenoids [62,63] that we would be observing in peak 3, tentatively identified as 15-apo-carotenal or the *m*/*z* 414 (peak 2), *m*/*z* 338 (peak 5) and *m*/*z* 457 (peak 7). The lack of identification of other carotenoids in the extract would also eventually be associated with degradation/oxidation processes. For caloxanthin (hydroxy-zeaxanthin) or its isomers there are no reports of their effects on human health, but the use of β-carotenetriol (=4-hydroxy-zeaxanthin) in fish feeding seems to enhance the metabolic production of astaxanthin, a carotenoid with antioxidant effects [64]. There is no information about the effects of 3,4-dihydrospirilloxanthin, hydroxyechinenone, and 15-apo-carotenal or their corresponding isomers on human health or as animal food.

The abundance of all these pigments in this microorganism may have evolved as a protective mechanism against harsh environmental conditions, including radiation levels and the low temperatures of the Antarctic region [65]. In fact, the synthesis of carotenoid pigments constitutes yet another antioxidant defense against oxidative stress, helps control the peroxidation rate of membrane lipids and also stabilizes membrane structure to assure the proper growth of bacteria in the extreme Antarctic environment [2,66]. This supports the idea that the pigments found in CN7 may counteract low temperatures and UV-mediated cellular stress.

Once isolated, carotenoids have several commercial applications. Some of those applications in mammals are geared towards their beneficial effect on diseases that affect skin and vision as a result of sun exposure [66]. There is a range of evidence to support a role for lutein and zeaxanthin in vision. Lutein and its isomer, zeaxanthin, are taken up selectively into eye tissue and may protect against eye disease, because they absorb damaging blue light that enters the eye. In addition, lutein and zeaxanthin in neural tissue may have biological effects that include antioxidation, anti-inflammation, and structural actions [67]. Another important application resides in the need for animal products for human consumption such as red meat, fish, and eggs, which in turn are accompanied by an upsurge in the production of improved quality products [68]. A cocktail of carotenoids is currently provided as an aquafeed to animals, birds, and fish to improve flesh coloration and is considered a safe practice [69,70]. For the aquaculture industry, which has grown exponentially in recent decades [71], fish quality, and thus market value, is closely related to flesh pigmentation [12]. Therefore, color is a decisive quality criterion that has to be maintained and optimized. Carotenoids may not only contribute to improving quality by enhancing color but could also help to create a better image of aquaculture products among consumers, in view of increasing information available on carotenoids positive effect on human health [72].

Accordingly, a bacterial strain that produces several carotenoid pigments, such as the CN7 Antarctic soil isolate, could be of great interest given the emphasis on the need for natural pigment coloring agents as an alternative to synthetic chemicals [73]. As the aquaculture feed industry seeks a natural, environmental friendly source of pigment to improve coloration and to enhance commercial acceptability, there is a great potential to use microorganism-based carotenoids for aquaculture pigmentation, paving the way for many aquaculture feed industries to promote their products as natural and free of synthetic ingredients and colorants [74,75]. 

## 5. Conclusions

The results of this study show that CN7, a psychrophilic bacterium isolated from Antarctica, produces a high diversity of carotenoid pigments, the most abundant being lutein and zeaxanthin. Given these properties, CN7 is a promising bacterial source of zeaxanthin, lutein, and lesser amounts of other natural carotenoids, such as capsanthin and echinenone. All of them have beneficial properties for human and animal health and are used as dietary supplements, particularly in the aquaculture industry.

## Figures and Tables

**Table 1:** Potential identification of carotenoid pigments in CN7 determined by LC-UV/vis-MS/MS.

Peak	RT (min)	Area (%)	Precursor (*m*/*z*)	Fragments MS^2^ (*m*/*z*)				Identification	References
1	15.7	0.4	585.2	492.3	566.4	525.4	485.6	Capsanthin	[31]
								Lutein-5,6-epoxide	[32]
								Caloxanthin (=Hydroxy-zeaxanthin)	[33]
2	17.2	0.7	584.4	492.2	564.4	567.0	477.2	Capsanthin	[34,35]
								Lutein-5,6-epoxide	[32]
								Caloxanthin (=Hydroxy-zeaxanthin)	[33]
			413.7	301.0	296.3	188.5	395.2	Not identified	
3	19.7	0.5	284.6	240.0	101.5	87.5	115.5	15-apo-carotenal	[36]
4	28.2	55.3	568.6	476.4	550.4	458.4	415.4	Lutein	[27,37,38]
								Zeaxanthin	[38,39,40]
5	32.1	37.6	621.7	381.3	515.4	399.3	529.4	Unknown	
			598.5	506.1	540.3	448.2	429.3	3,4-dihydrospirilloxanthin *	[41]
			338.4	321.2	303.2	162.7		Unknown	
6	34.2	2.5	598.6	506.0	540.3	448.2	429.3	3,4-dihydrospirilloxanthin *	[41]
			568.4	476.3	549.4	546.5	509.5	Lutein	[27,37,38]
								Zeaxanthin	[38,39,40]
7	37.3	3.0	598.7	540.3	506.3	429.3	448.1	3,4-dihydrospirilloxanthin *	[41]
			567.8	550.5	509.6	529.8	461.3	Hydroxyechinenone	[33]
			457.3	345.1	232.7	398.3	438.2	Unknown	

Note: compounds marked with an asterisk were further identified in the MassBank database.

## Supplementary Materials

The following are available online at https://www.mdpi.com/article/10.3390/microorganisms9122419/s1, Figure S1: Mass spectrum of Peak 1, Figure S2: MS/MS spectra of *m/z* 585 (Peak 1). The assignment of some *m/z* signals are shown in red, Figure S3: Interpretation of MS/MS spectra of *m/z* 585 (Peak 1). The structure of the caloxanthin is shown, on this the fragmentation sites that would explain the *m/z* signals observed in the MS/MS spectrum are outlined. The loss of 60 Da (elimination of 1,2-ethenediol) would correspond to a retro Diels-Alder reaction in β-ring, Figure S4: Mass spectrum of Peak 2, Figure S5: MS/MS spectra of *m/z* 584 (Peak 2). The assignment of some *m/z* signals are shown in red, Figure S6: Interpretation of MS/MS spectra of *m/z* 584 (Peak 2). The structure of the caloxanthin is shown, on this the fragmentation sites that would explain the *m/z* signals observed in the MS/MS spectrum are outlined. The loss of 60 Da (elimination of 1,2-ethenediol) would correspond to a retro Diels-Alder reaction in β-ring, Figure S7: MS/MS spectra of *m/z* 413 (Peak 2), Figure S8: Mass spectrum of Peak 3, Figure S9: MS/MS spectra of *m/z* 284 (Peak 3). The assignment of some *m/z* signals are shown in red, Figure S10: Interpretation of MS/MS spectra of *m/z* 284 (Peak 3). Based on the structure of 15-apo-carotenal (left) some fragments are outlined, the keto group would be lost as formaldehyde and the free end would then begin to fragment producing C_3_H_4_ or C_2_H_2_. It is also possible that fragmentation may occur in the β-ring through a retro Diels-Alder reaction to produce C_2_H_2_. Additionally, a balance was postulated between the keto form (left) and an alcohol (right) that would explain the elimination of water, from this end C_2_H_2_ would be lost, Figure S11: Mass spectrum of Peak 4, Figure S12: MS/MS spectra of *m/z* 568 (Peak 4). The assignment of some *m/z* signals are shown in red, Figure S13: Interpretation of MS/MS spectra of *m/z* 568 (Peak 4). The structure of the zeaxanthin is shown, on this the fragmentation sites that would explain the *m/z* signals observed in the MS/MS spectrum are outlined. The presence of zeaxanthin would be confirmed by the low intensity of the signal *m/z* 551 and the absence of *m/z* 495 according to what has been described in the literature; additionally, the %III/II coincides with that described for zeaxanthin, Figure S14: Mass spectrum of Peak 5, Figure S15: MS/MS spectra of *m/z* 621 (Peak 5). The assignment of some *m/z* signals are shown in red, Figure S16: MS/MS spectra of *m/z* 598 (Peak 5). The assignment of some *m/z* signals are shown in red, Figure S17: Interpretation of MS/MS spectra of *m/z* 598 (Peak 5). The structure of the 3,4-dihydrospirilloxanthin is shown, on this the fragmentation sites that would explain the *m/z* signals observed in the MS/MS spectrum are outlined. The presence of methoxy groups was observed through the loss of methanol (CH3OH) or formaldehyde (CH_2_ = O), the elimination of methyl was not observed, Figure S18: Mass spectrum of Peak 6, Figure S19: MS/MS spectra of *m/z* 598 (Peak 6). The assignment of some *m/z* signals are shown in red, Figure S20: Interpretation of MS/MS spectra of *m/z* 598 (Peak 6). The structure of the 3,4-dihydrospirilloxanthin is shown, on this the fragmentation sites that would explain the *m/z* signals observed in the MS/MS spectrum are outlined. The presence of methoxy groups was observed through the loss of methanol (CH_3_OH) or formaldehyde (CH_2_ = O), the elimination of methyl was not observed, Figure S21: MS/MS spectra of *m/z* 568 (Peak 6). The assignment of some *m/z* signals are shown in red, Figure S22: Interpretation of MS/MS spectra of *m/z* 568 (Peak 6). The structure of the lutein is shown, on this the fragmentation sites that would explain the *m/z* signals observed in the MS/MS spectrum are outlined. The presence of lutein would be confirmed by the high intensity of the signal *m/z* 551 and the presence of *m/z* 495 according to what has been described in the literature; additionally, the %III/II coincides with that described for lutein, Figure S23: Mass spectrum of Peak 7, Figure S24: MS/MS spectra of *m/z* 598 (Peak 7). The assignment of some *m/z* signals are shown in red, Figure S25: Interpretation of MS/MS spectra of *m/z* 598 (Peak 7). The structure of the 3,4-dihydrospirilloxanthin is shown, on this the fragmentation sites that would explain the *m/z* signals observed in the MS/MS spectrum are outlined. The presence of methoxy groups was observed through the loss of methanol (CH3OH) or formaldehyde (CH_2_ = O), the elimination of methyl was not observed, Figure S26: MS/MS spectra of *m/z* 567 (Peak 7). The assignment of some *m/z* signals are shown in red, Figure S27: Interpretation of MS/MS spectra of *m/z* 567 (Peak 7). The structure of the hydroxyechinenone is shown, on this the fragmentation sites that would explain the *m/z* signals observed in the MS/MS spectrum are outlined, Figure S28: MS/MS spectra of *m/z* 457 (Peak 7).
