# Supplementary material for: Carotenoid Cocktail Produced by An Antarctic Soil Flavobacterium with Biotechnological Potential
Source: Microorganisms. 2021 Nov 24;9(12):2419. doi: 10.3390/microorganisms9122419 (PMC8704924; doi:10.3390/microorganisms9122419)
Supplement: Supplementary file 1 [file microorganisms-09-02419-s001.zip › microorganisms-1368113-supplementary.pdf]

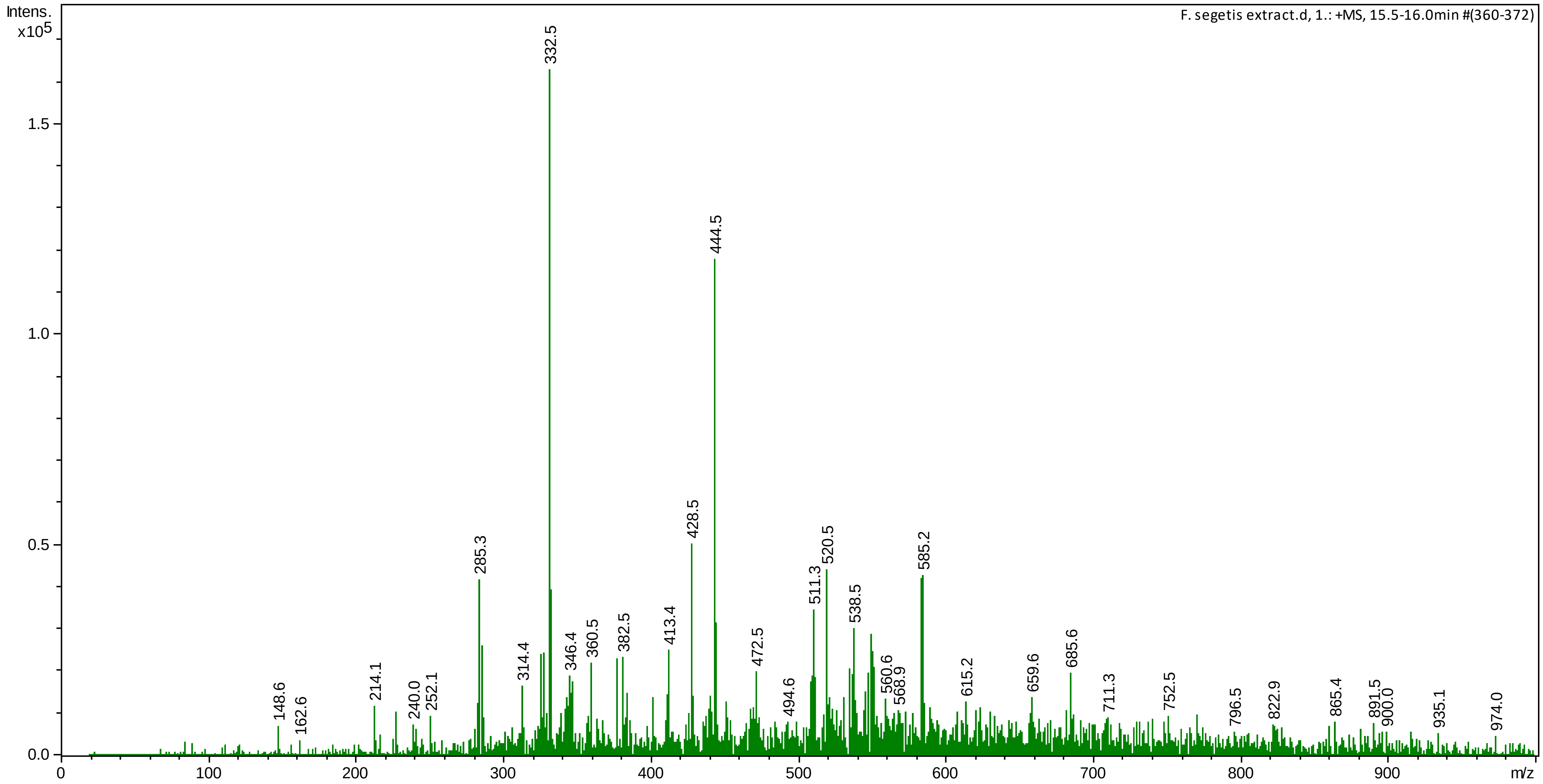

Figure S1. Mass spectrum of Peak 1.

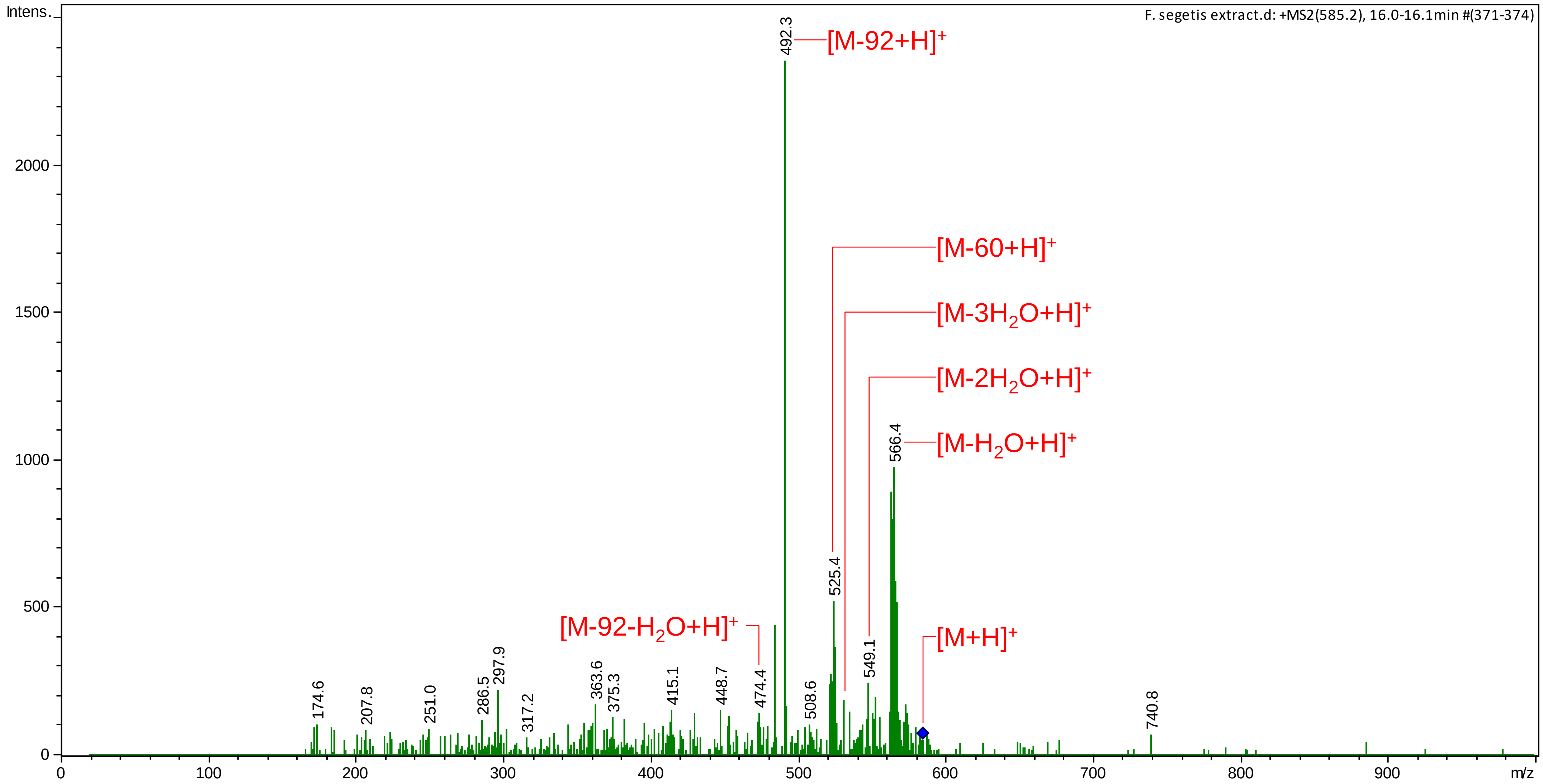

Figure S2. MS/MS spectra of m/z 585 (Peak 1). The assignment of some m/z signals are shown in red.

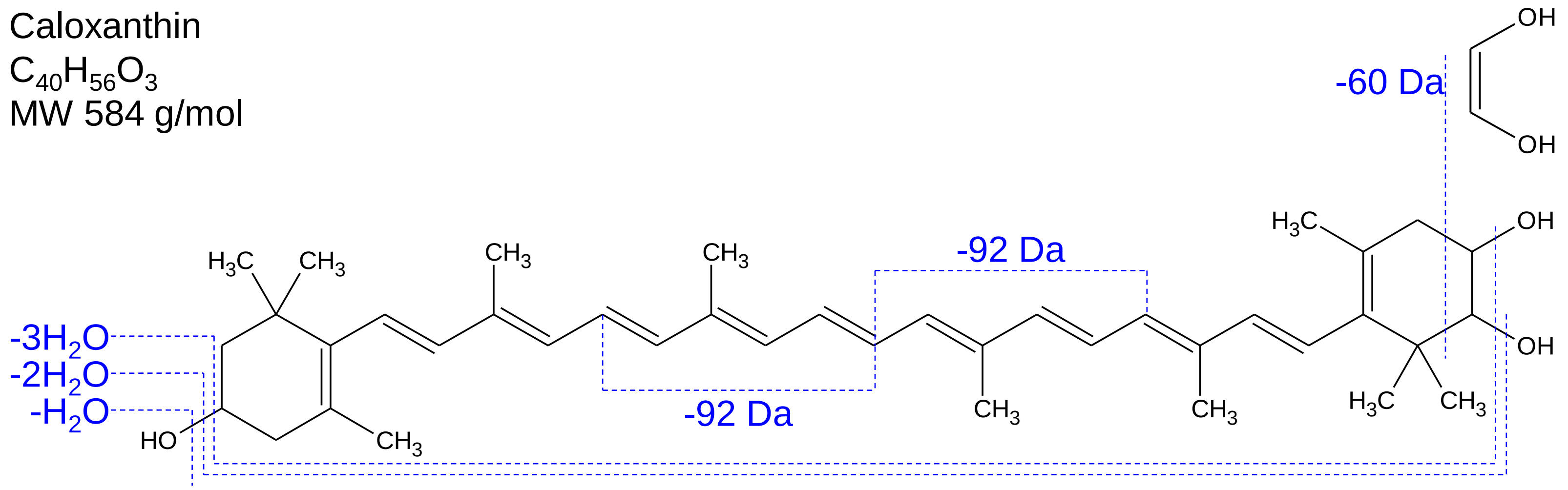

Figure S3. Interpretation of MS/MS spectra of m/z 585 (Peak 1). The structure of the caloxanthin is shown, on this the fragmentation sites that would explain the m/z signals observed in the MS/MS spectrum are outlined. The loss of 60 Da (elimination of 1,2-ethenediol) would correspond to a retro Diels-Alder reaction in β-ring.

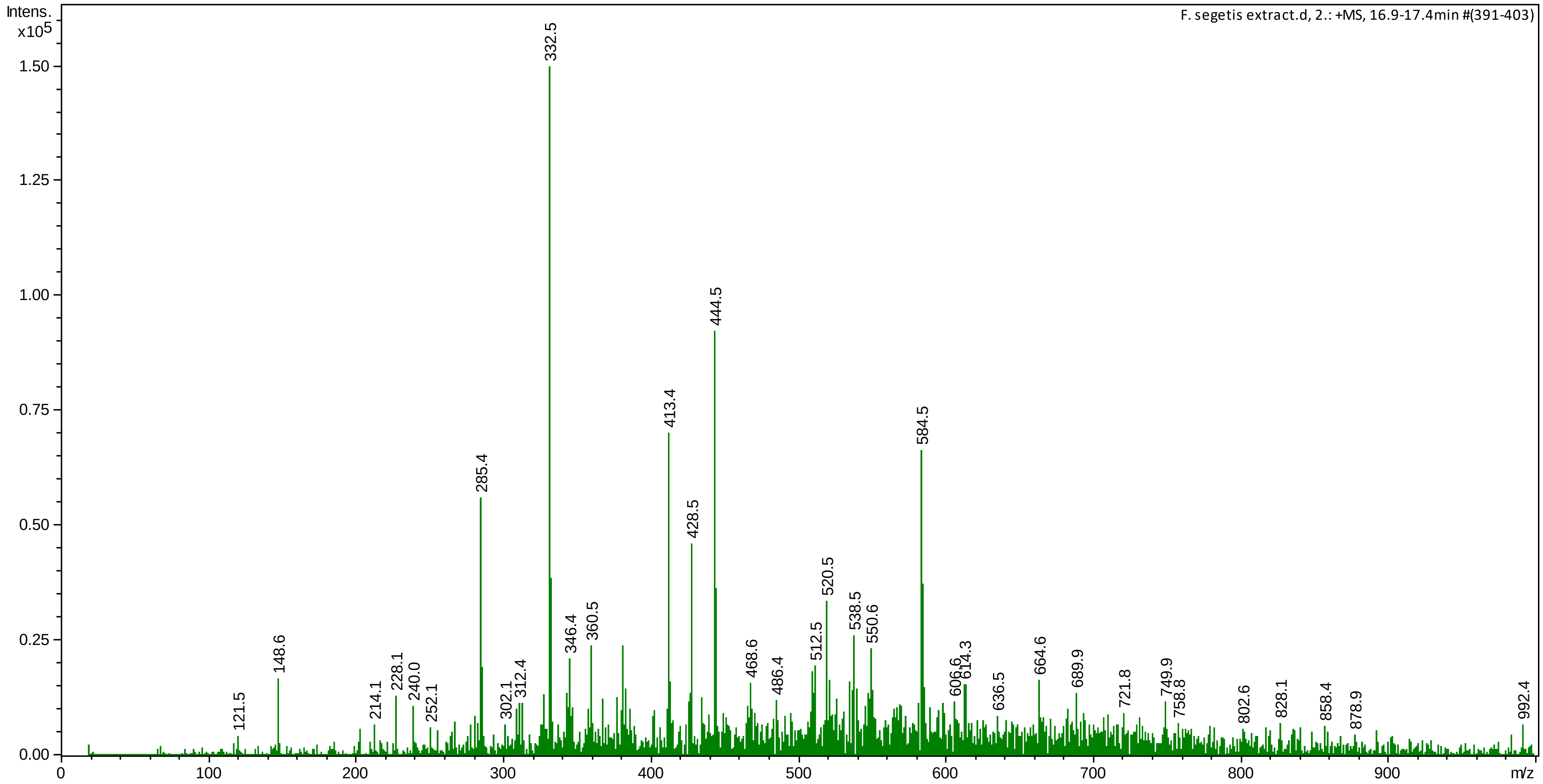

Figure S4. Mass spectrum of Peak 2.

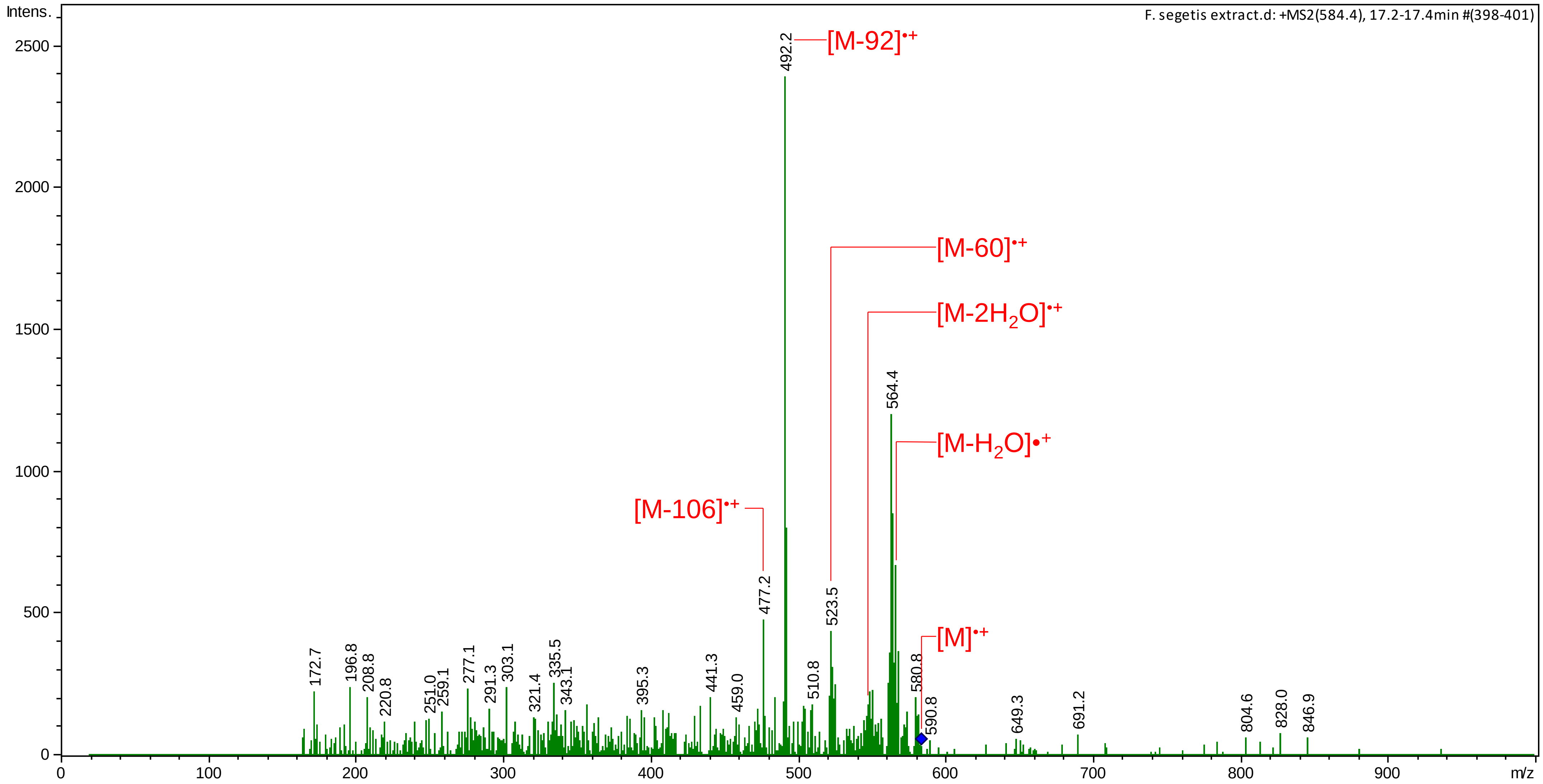

Figure S5. MS/MS spectra of m/z 584 (Peak 2). The assignment of some m/z signals are shown in red.

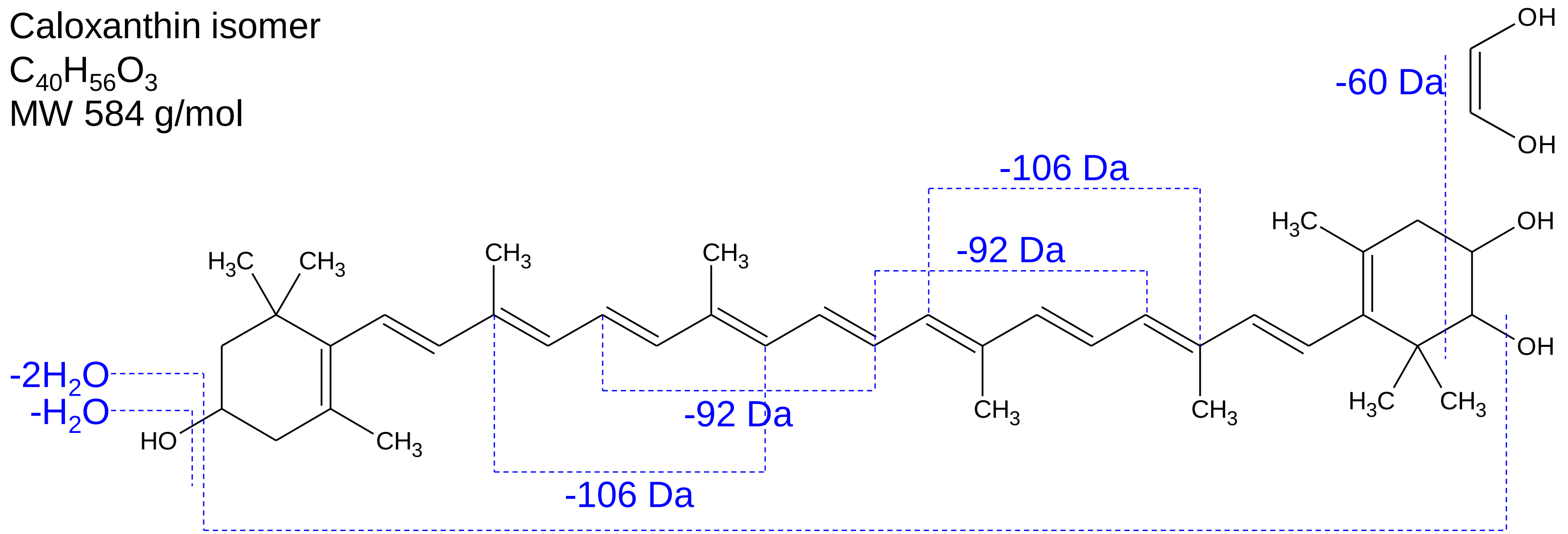

Figure S6. Interpretation of MS/MS spectra of m/z 584 (Peak 2). The structure of the caloxanthin is shown, on this the fragmentation sites that would explain the m/z signals observed in the MS/MS spectrum are outlined. The loss of 60 Da (elimination of 1,2-ethenediol) would correspond to a retro Diels-Alder reaction in β-ring.

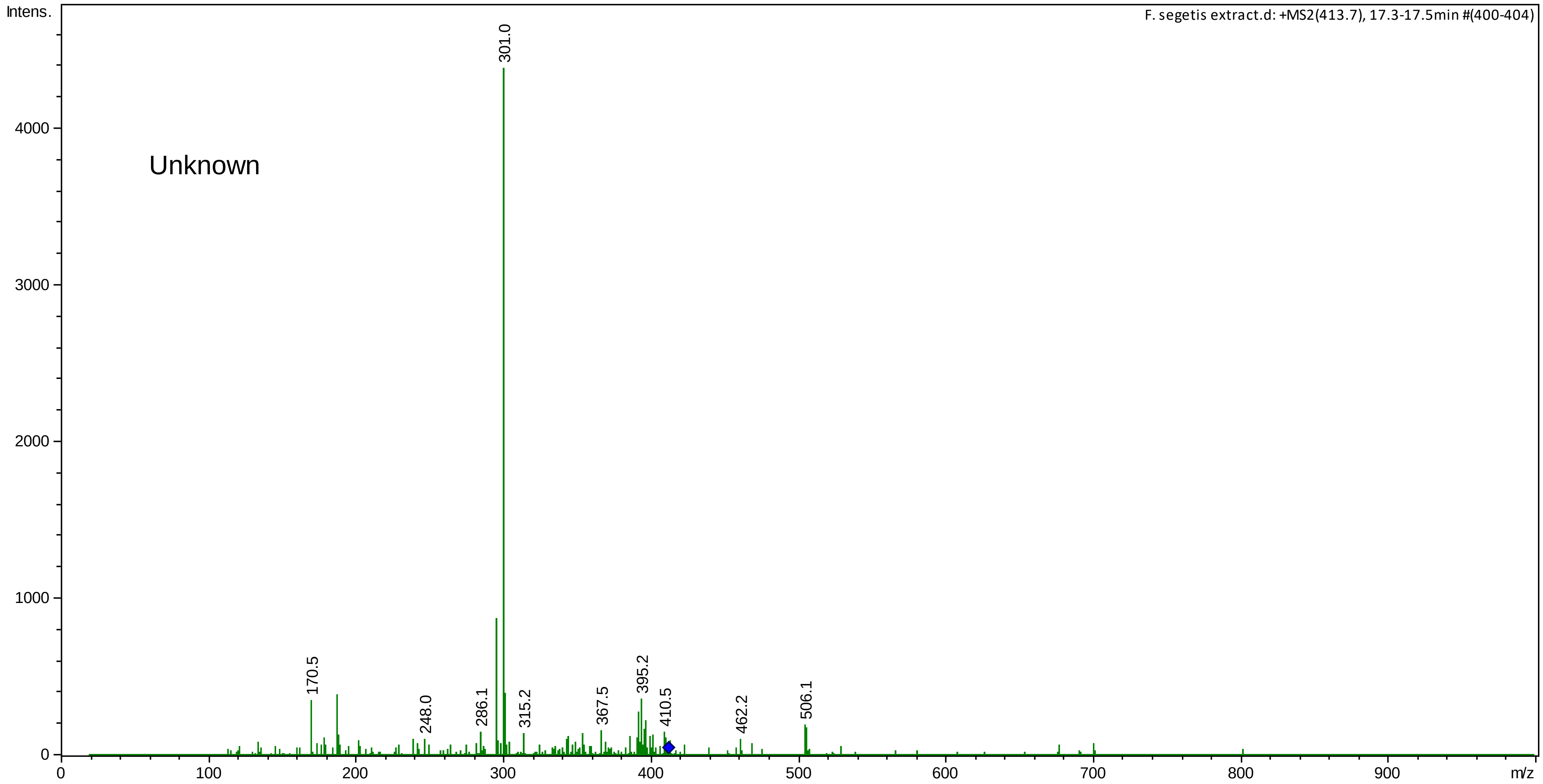

Figure S7. MS/MS spectra of m/z 413 (Peak 2).

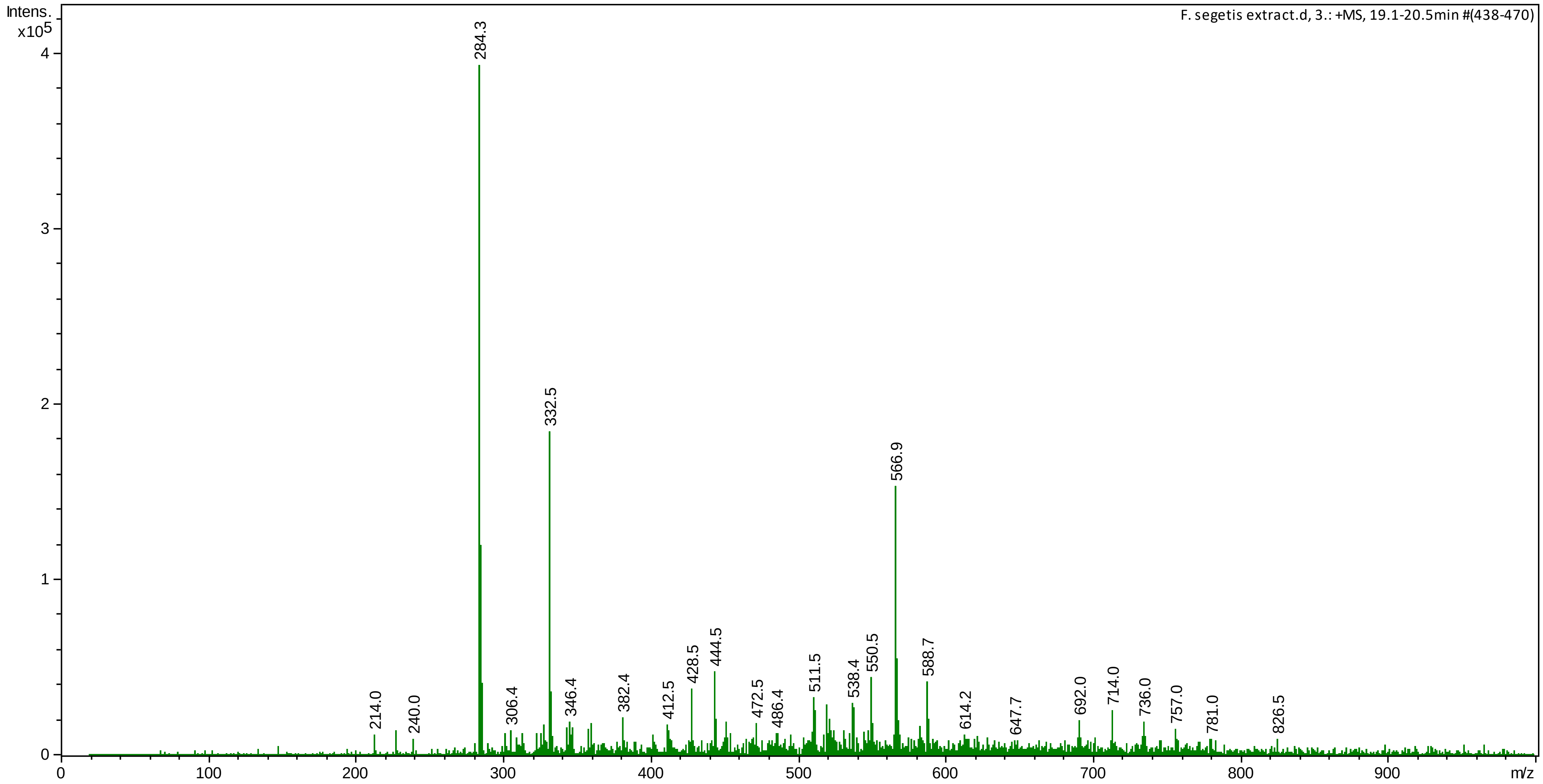

Figure S8. Mass spectrum of Peak 3.

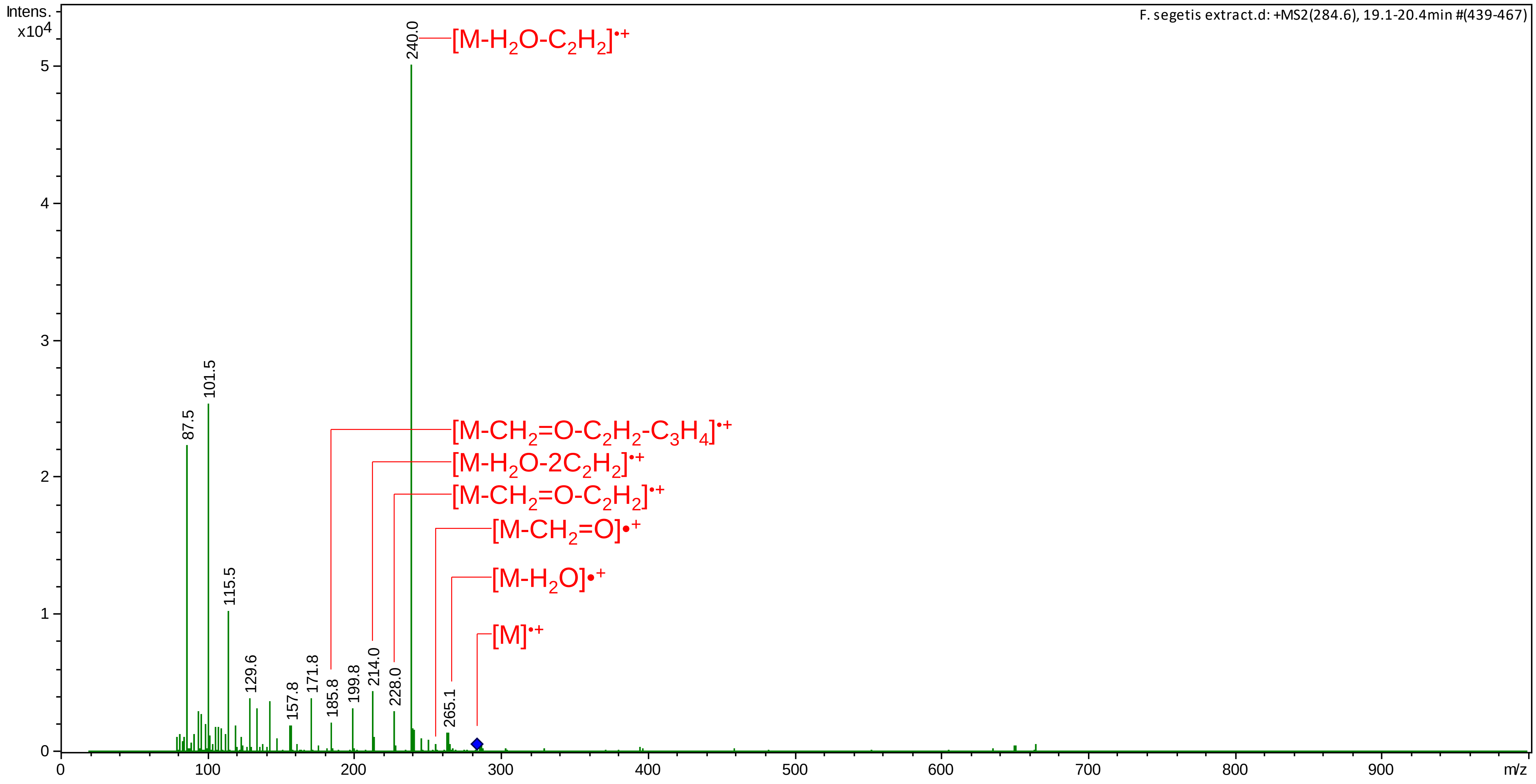

Figure S9. MS/MS spectra of m/z 284 (Peak 3). The assignment of some m/z signals are shown in red.

15-apo-carotenal
$C_{20}H_{28}O$
MW 284 g/mol

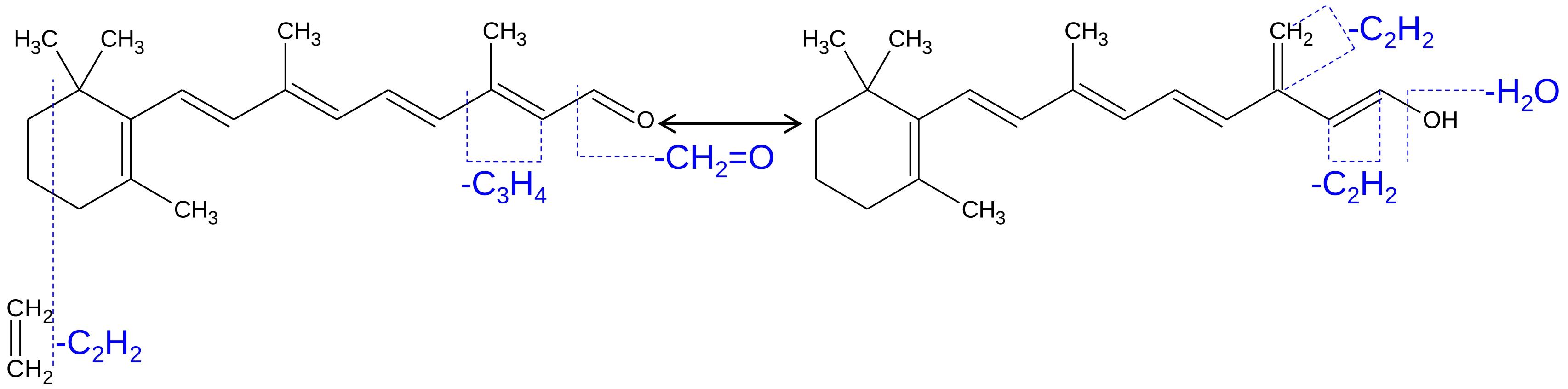

Figure S10. Interpretation of MS/MS spectra of m/z 284 (Peak 3). Based on the structure of 15-apo-carotenal (left) some fragments are outlined, the keto group would be lost as formaldehyde and the free end would then begin to fragment producing C_3H_4 or C_2H_2. It is also possible that fragmentation may occur in the β-ring through a retro Diels-Alder reaction to produce C_2H_2. Additionally, a balance was postulated between the keto form (left) and an alcohol (right) that would explain the elimination of water, from this end C_2H_2 would be lost.

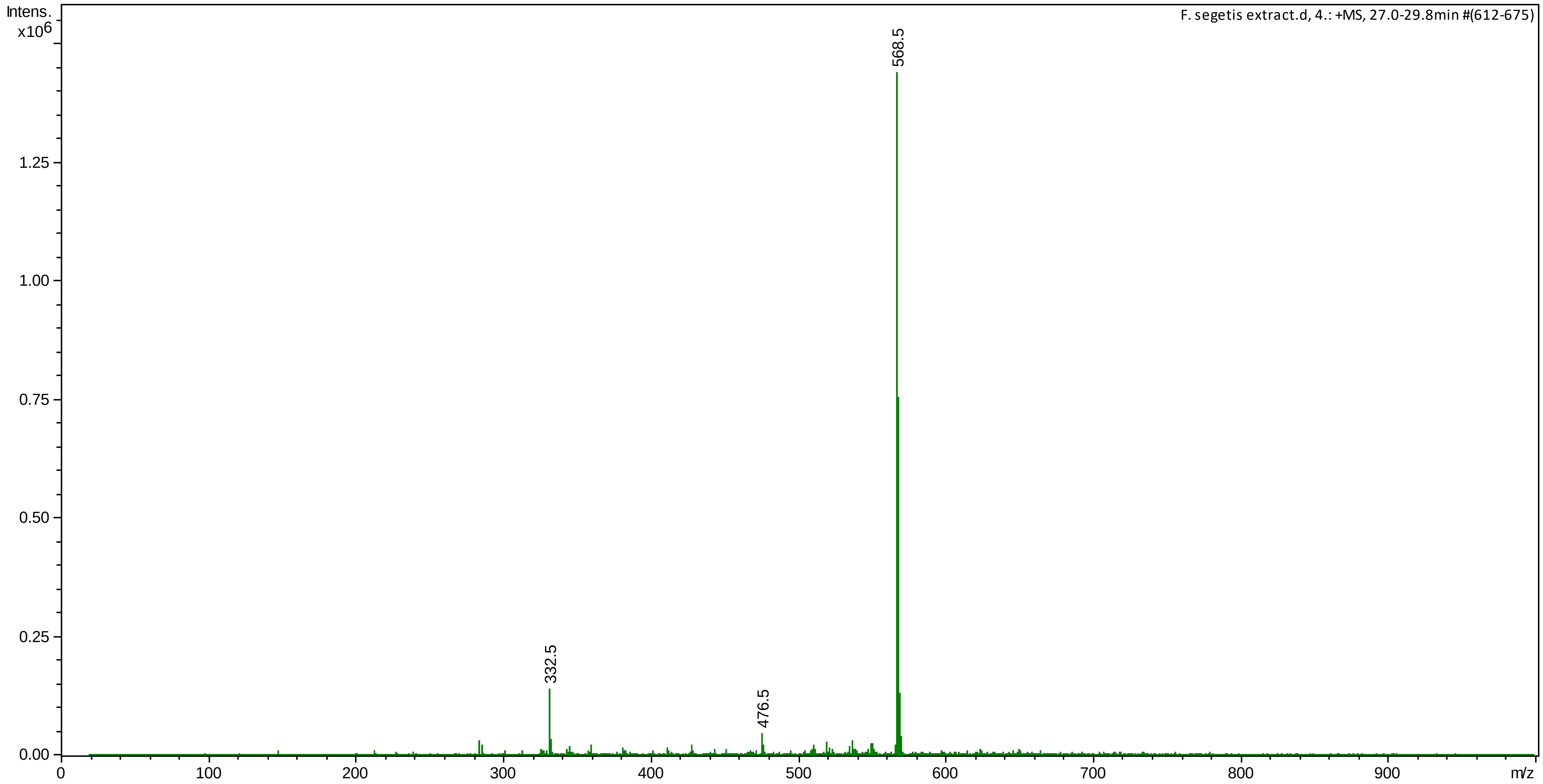

Figure S11. Mass spectrum of Peak 4.

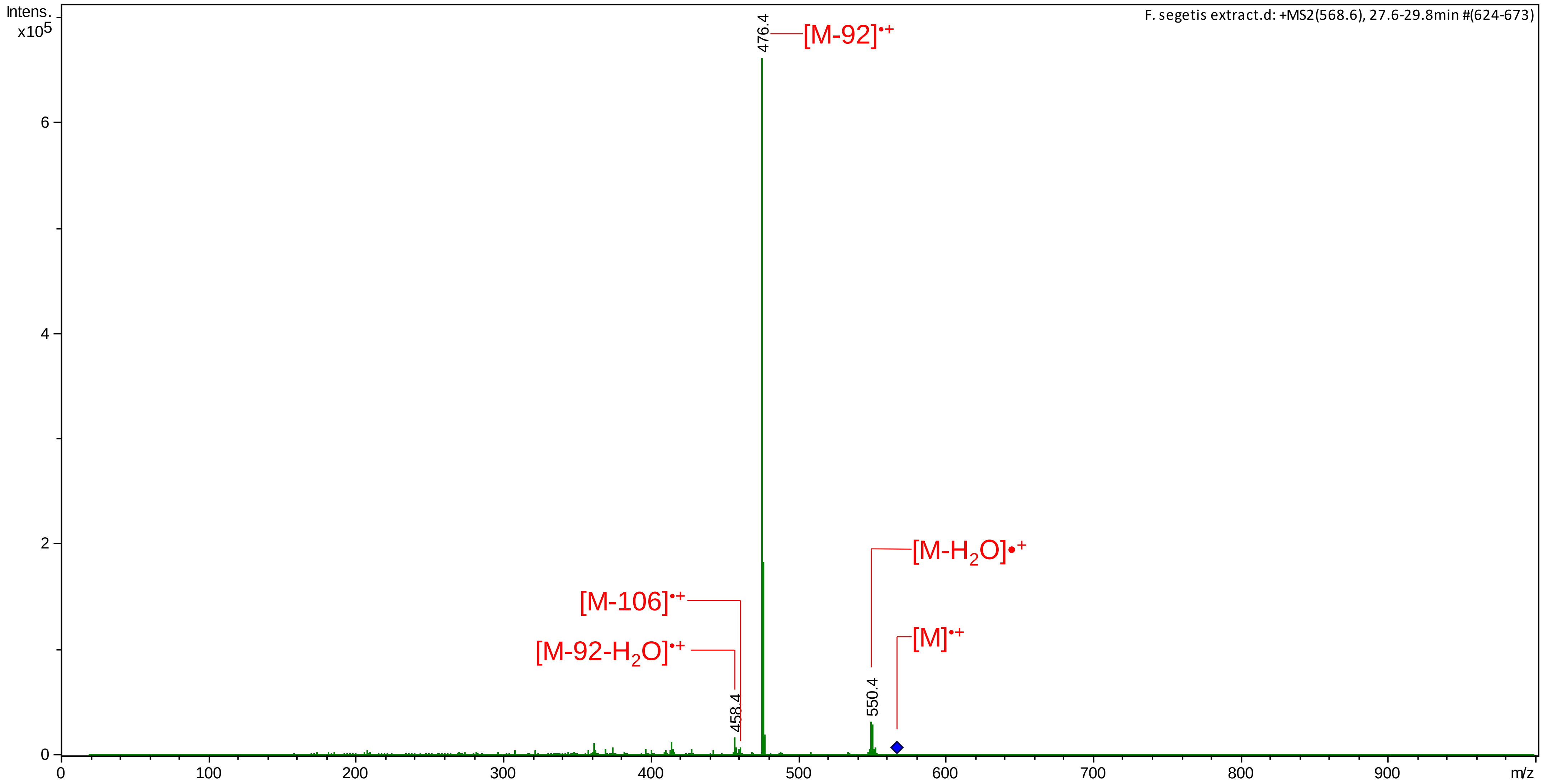

Figure S12. MS/MS spectra of m/z 568 (Peak 4). The assignment of some m/z signals are shown in red.

Zeaxanthin/(Lutein)
$C_{40}H_{56}O_2$
MW 568 g/mol

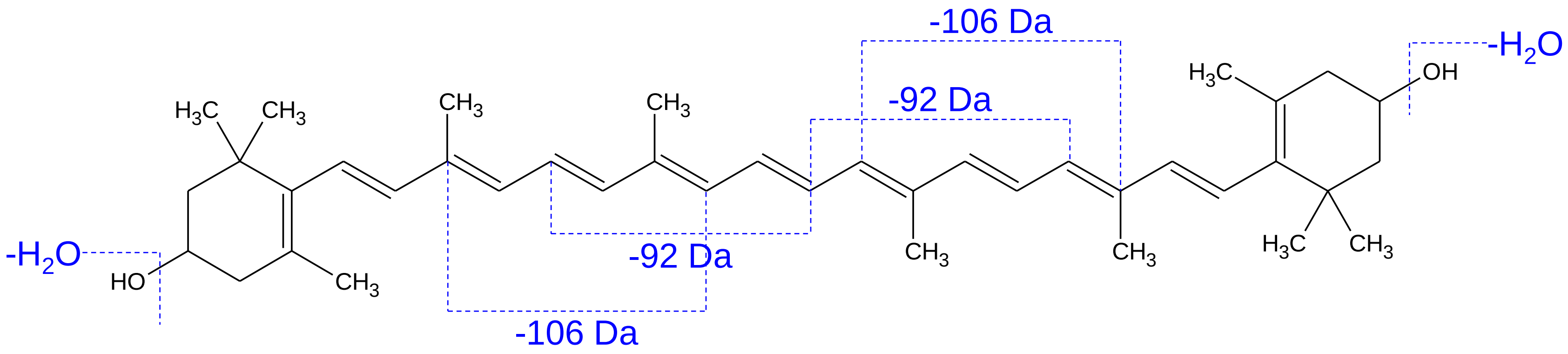

Figure S13. Interpretation of MS/MS spectra of m/z 568 (Peak 4). The structure of the zeaxanthin is shown, on this the fragmentation sites that would explain the m/z signals observed in the MS/MS spectrum are outlined. The presence of zeaxanthin would be confirmed by the low intensity of the signal m/z 551 and the absence of m/z 495 according to what has been described in the literature; additionally, the %III/II coincides with that described for zeaxanthin.

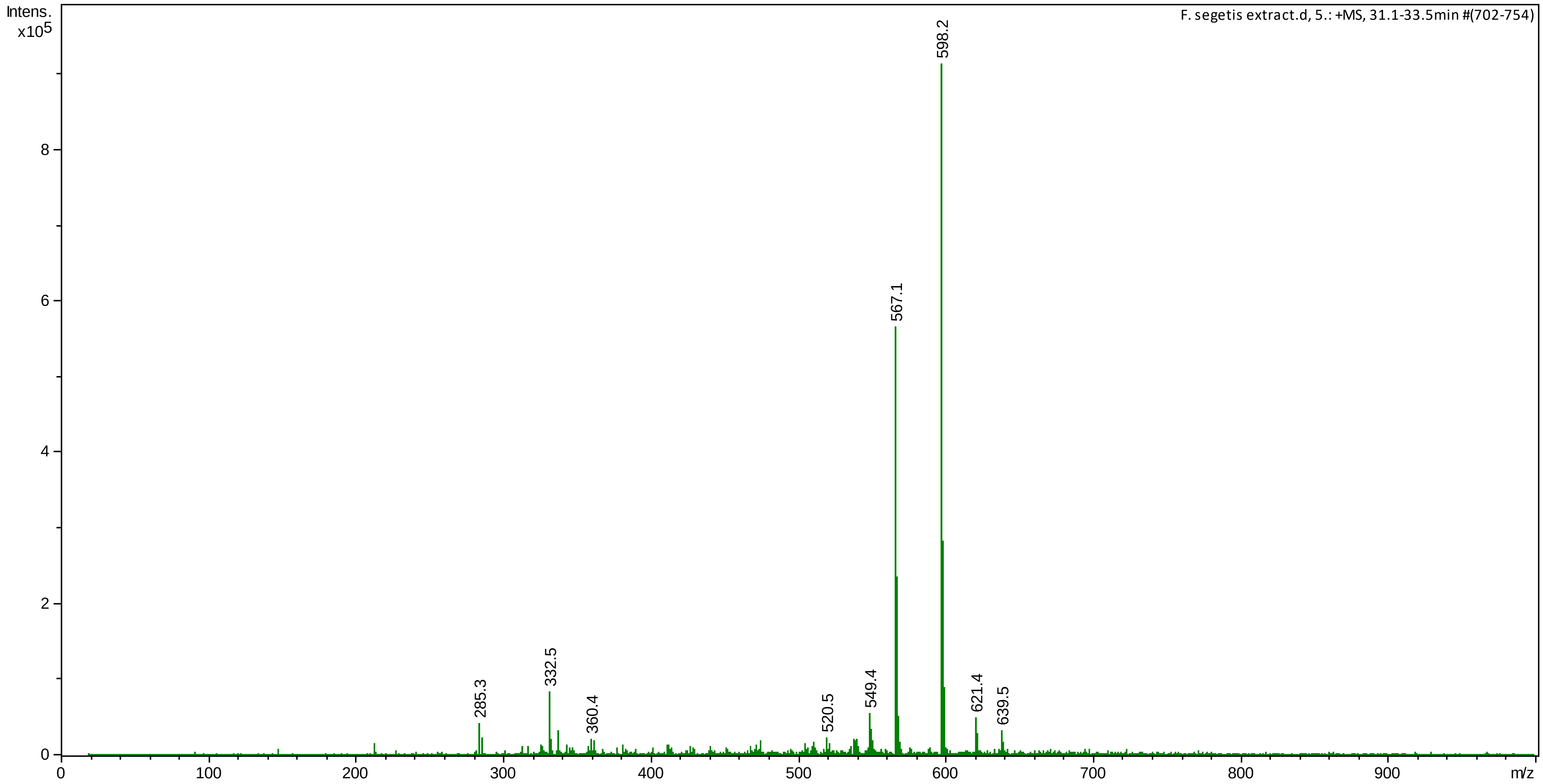

Figure S14. Mass spectrum of Peak 5.

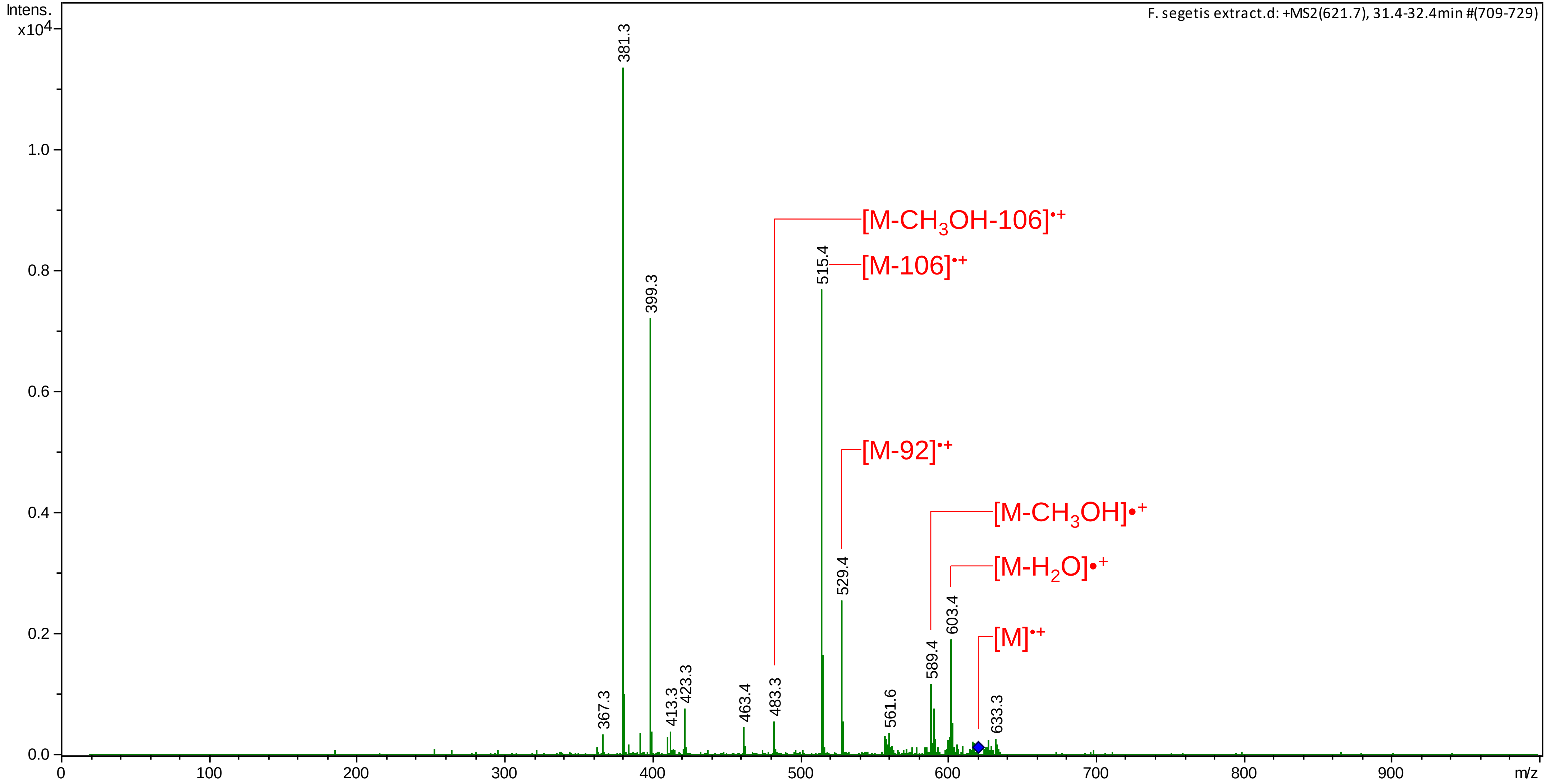

Figure S15. MS/MS spectra of m/z 621 (Peak 5). The assignment of some m/z signals are shown in red.

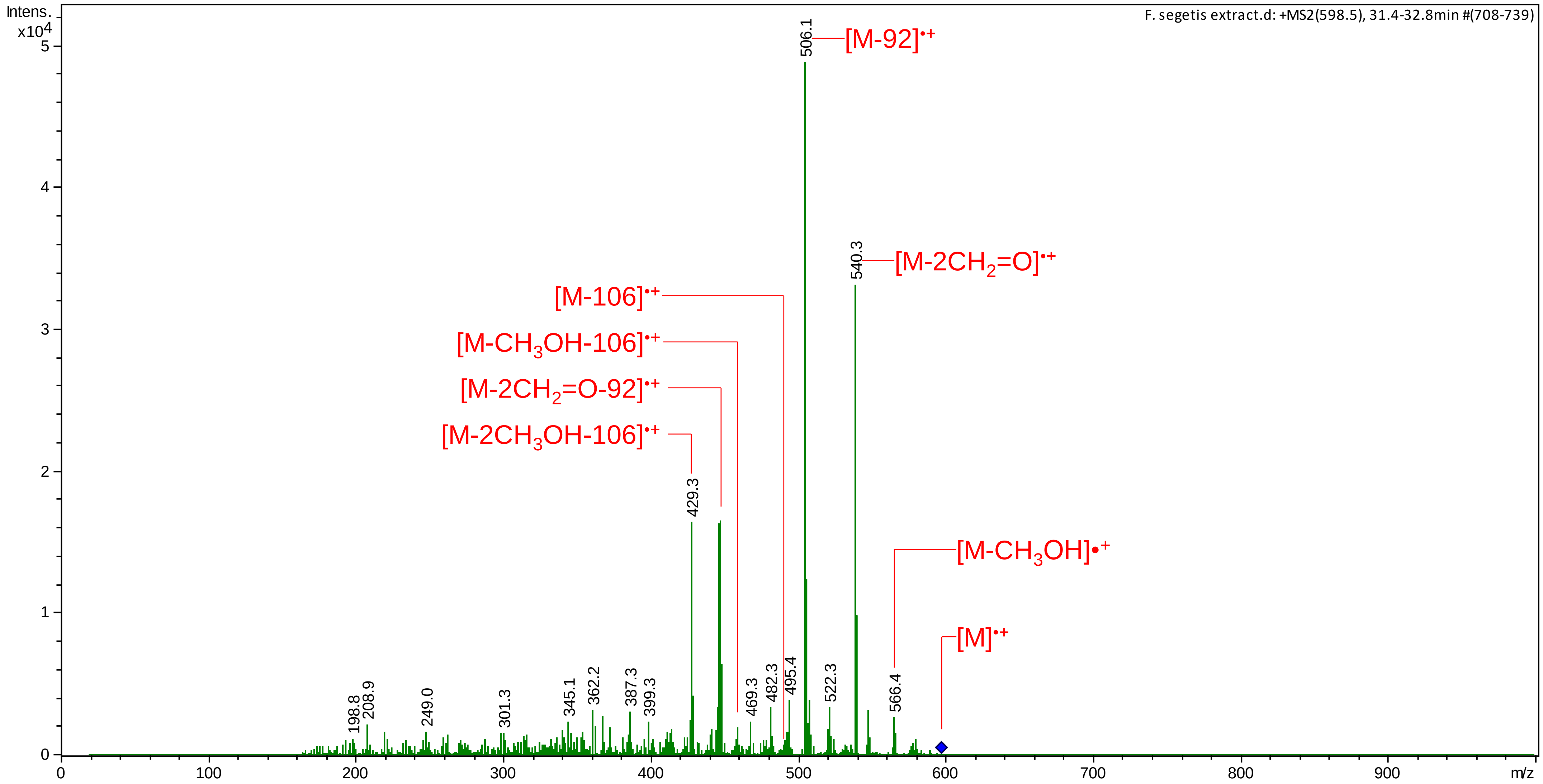

Figure S16. MS/MS spectra of m/z 598 (Peak 5). The assignment of some m/z signals are shown in red.

3,4-dihydrospirilloxanthin
$C_{40}H_{62}O_2$
MW 598 g/mol

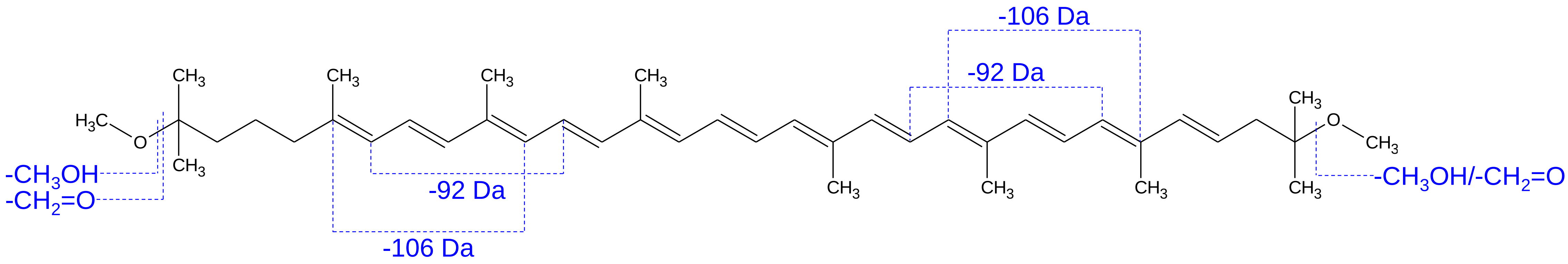

Figure S17. Interpretation of MS/MS spectra of m/z 598 (Peak 5). The structure of the 3,4-dihydrospirilloxanthin is shown, on this the fragmentation sites that would explain the m/z signals observed in the MS/MS spectrum are outlined. The presence of methoxy groups was observed through the loss of methanol (CH_3OH) or formaldehyde (CH_2=O), the elimination of methyl was not observed.

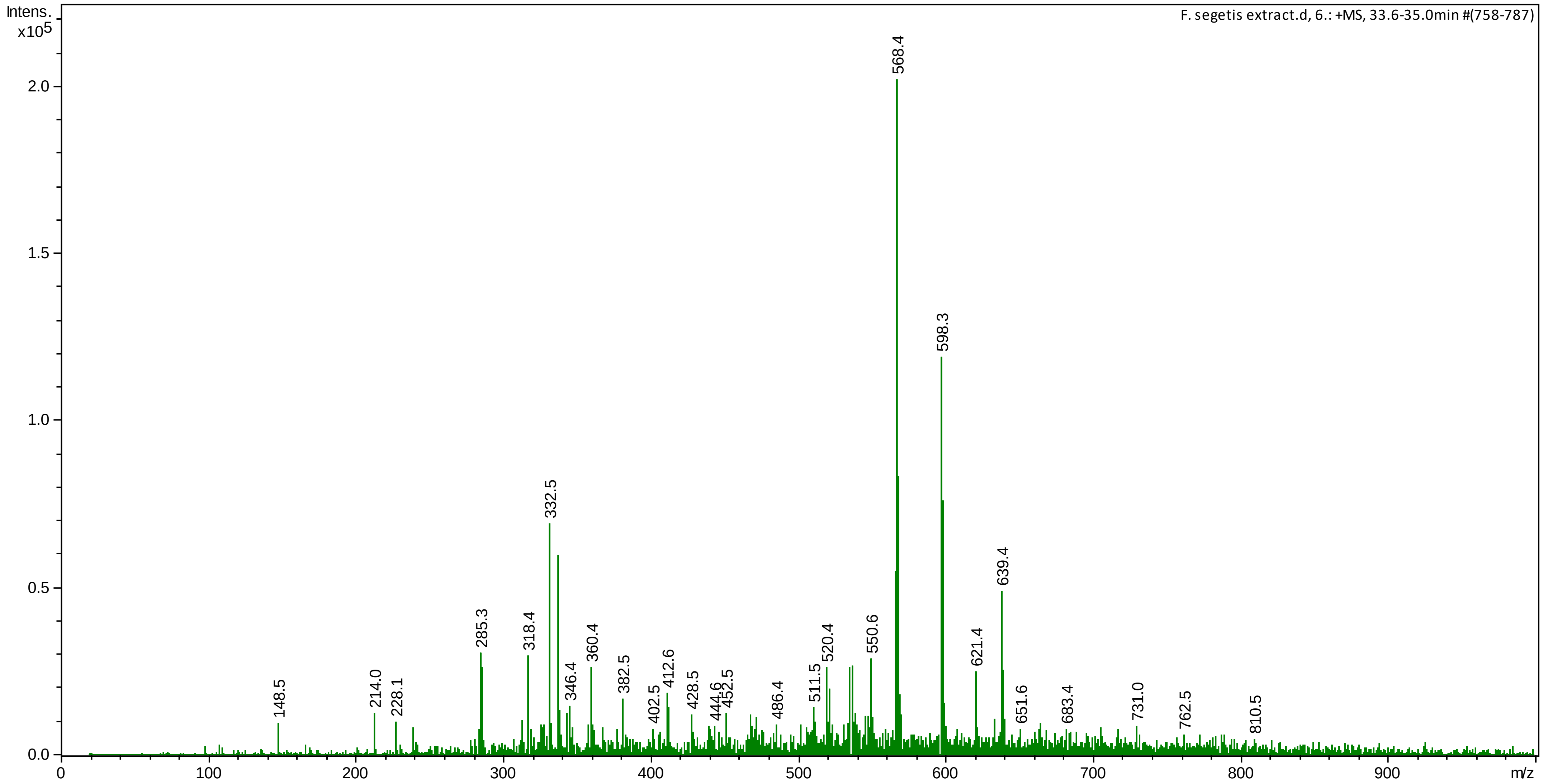

Figure S18. Mass spectrum of Peak 6.

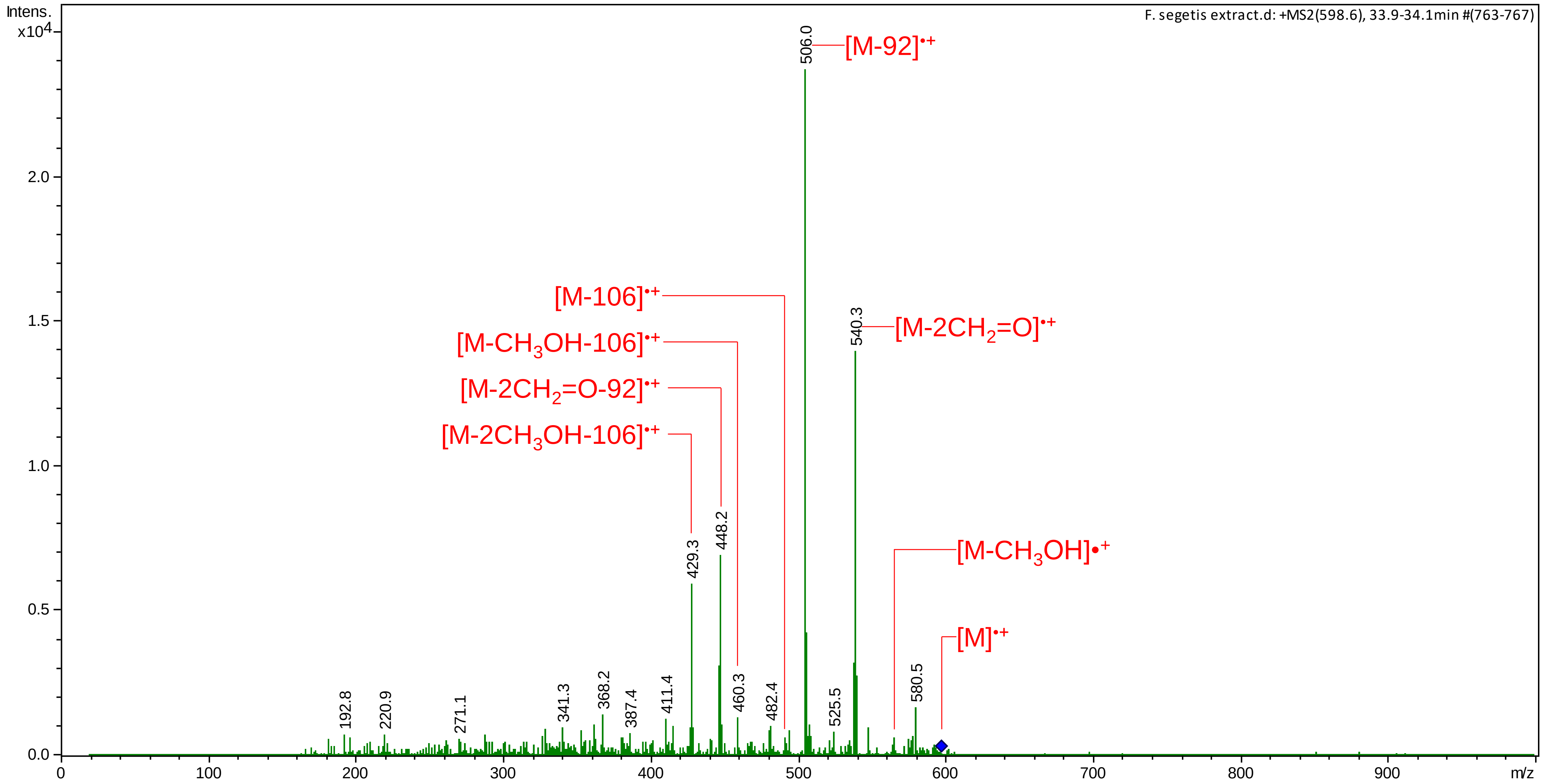

Figure S19. MS/MS spectra of m/z 598 (Peak 6). The assignment of some m/z signals are shown in red.

3,4-dihydrospirilloxanthin isomer
$C_{40}H_{62}O_2$
MW 598 g/mol

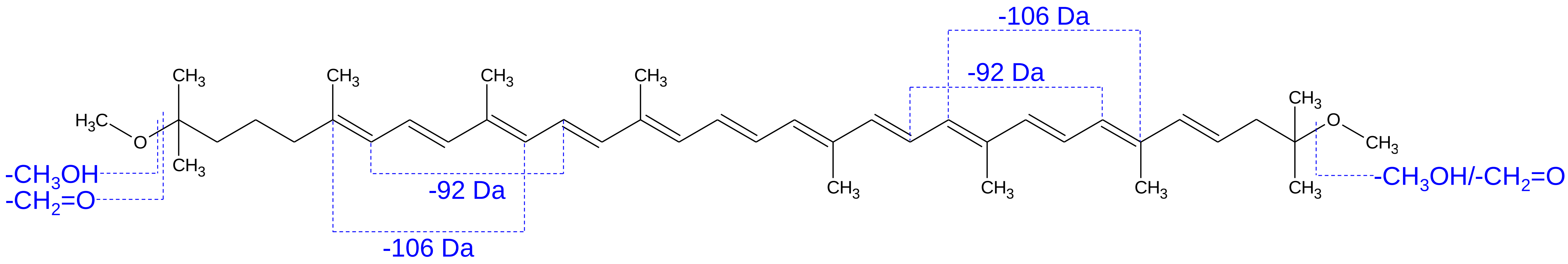

Figure S20. Interpretation of MS/MS spectra of m/z 598 (Peak 6). The structure of the 3,4-dihydrospirilloxanthin is shown, on this the fragmentation sites that would explain the m/z signals observed in the MS/MS spectrum are outlined. The presence of methoxy groups was observed through the loss of methanol (CH_3OH) or formaldehyde (CH_2=O), the elimination of methyl was not observed.

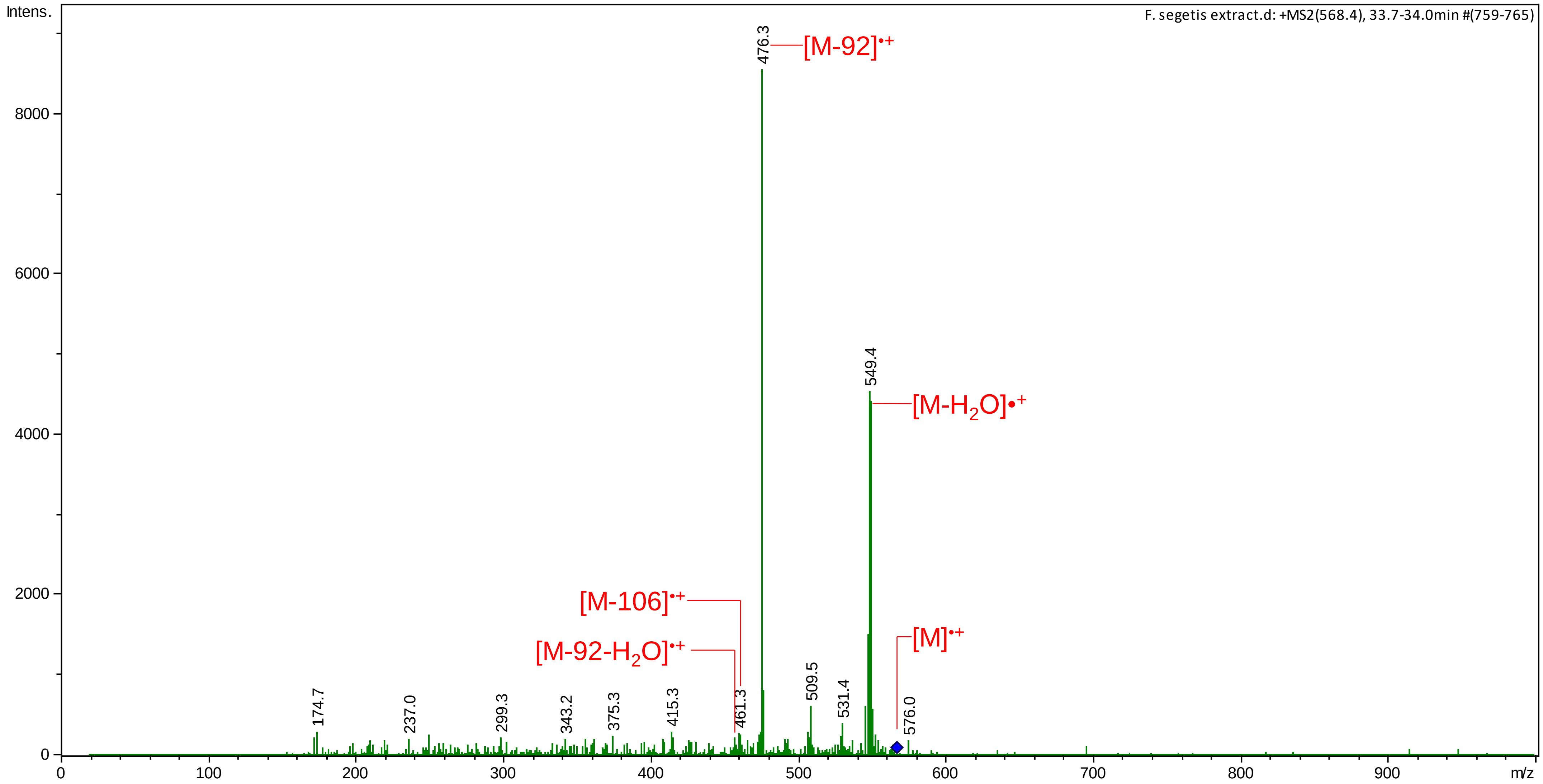

Figure S21. MS/MS spectra of m/z 568 (Peak 6). The assignment of some m/z signals are shown in red.

Lutein/(Zeaxanthin)
$C_{40}H_{56}O_2$
MW 568 g/mol

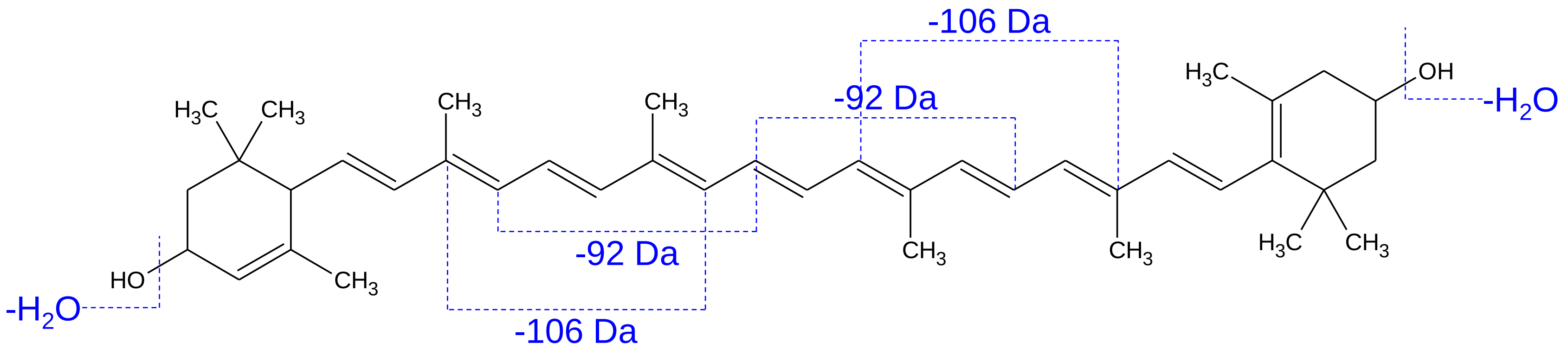

Figure S22. Interpretation of MS/MS spectra of m/z 568 (Peak 6). The structure of the lutein is shown, on this the fragmentation sites that would explain the m/z signals observed in the MS/MS spectrum are outlined. The presence of lutein would be confirmed by the high intensity of the signal m/z 551 and the presence of m/z 495 according to what has been described in the literature; additionally, the %III/II coincides with that described for lutein.

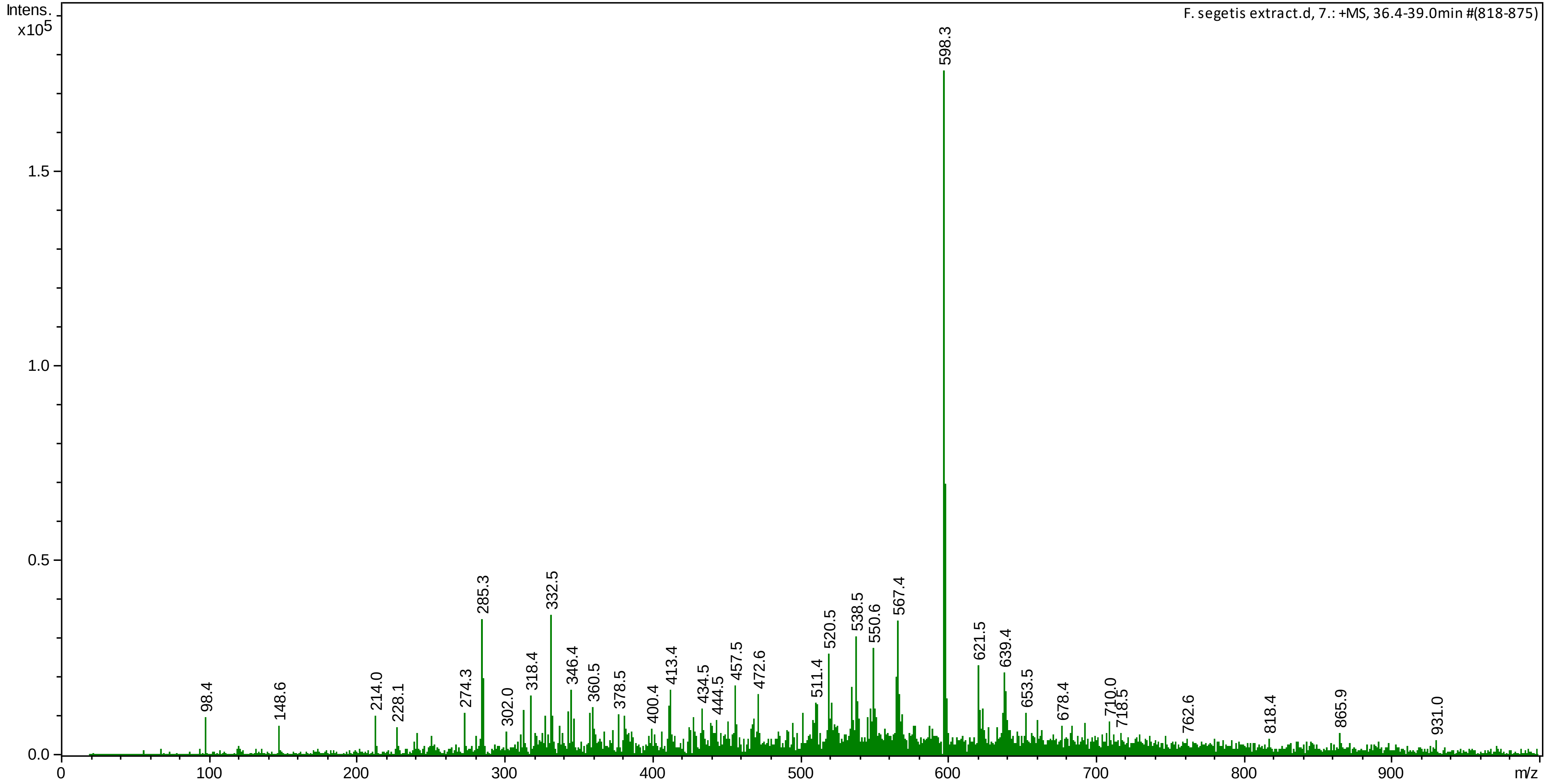

Figure S23. Mass spectrum of Peak 7.

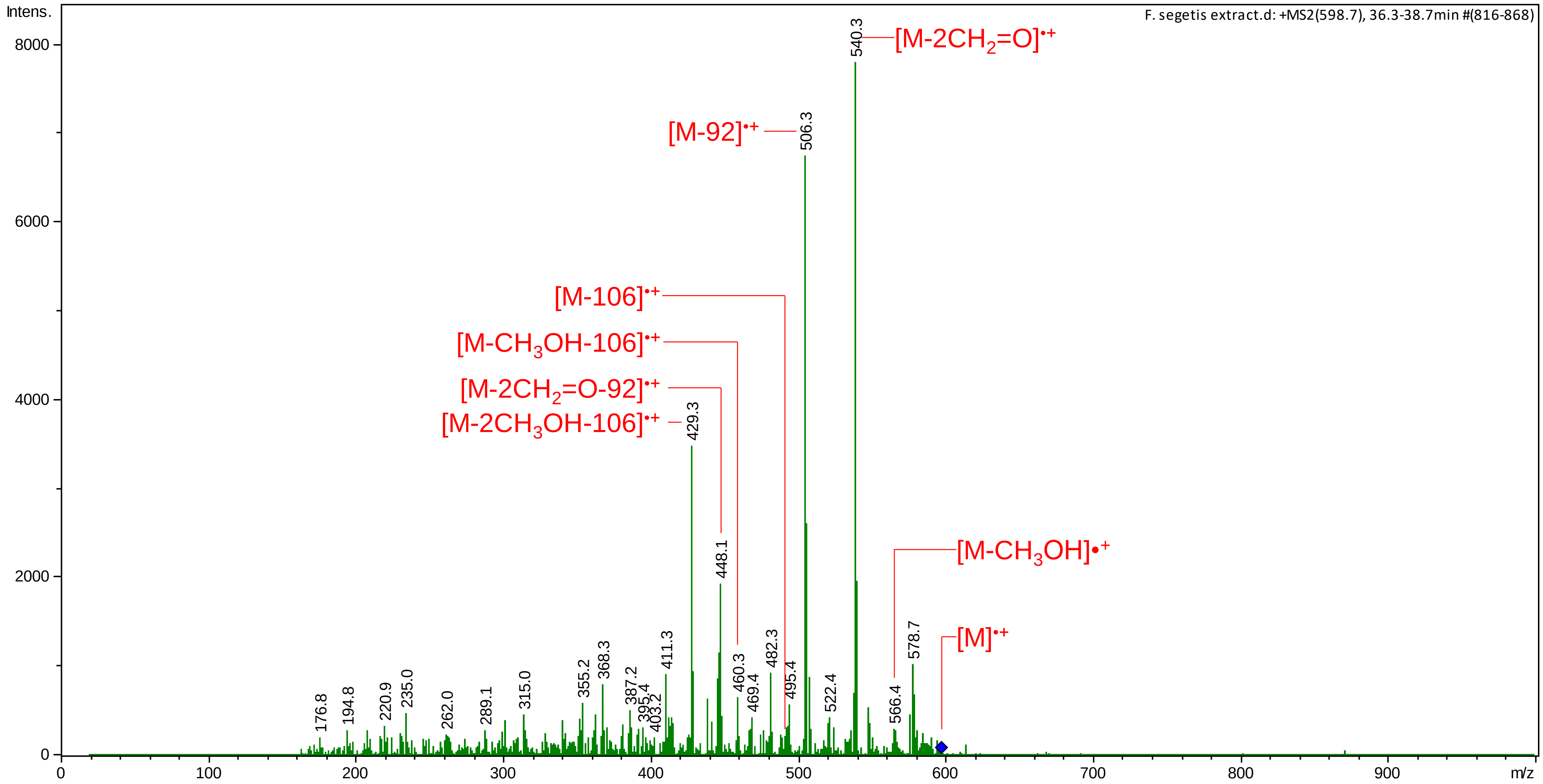

Figure S24. MS/MS spectra of m/z 598 (Peak 7). The assignment of some m/z signals are shown in red.

3,4-dihydrospirilloxanthin isomer
$C_{40}H_{62}O_2$
MW 598 g/mol

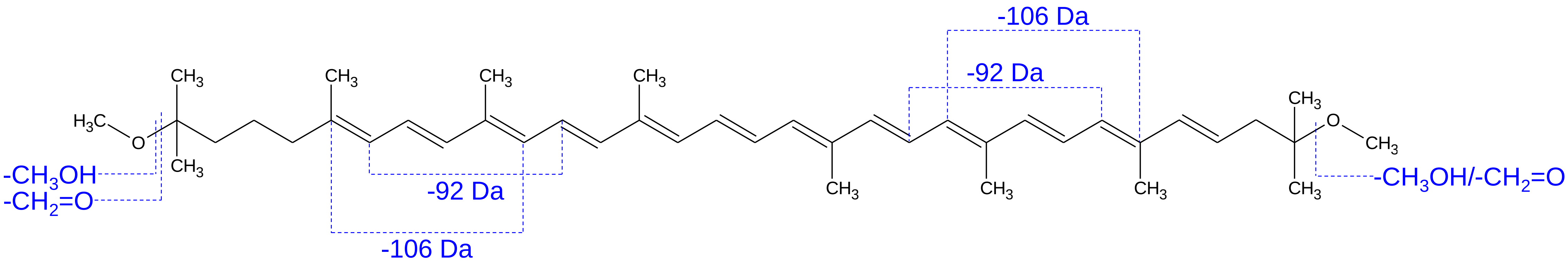

Figure S25. Interpretation of MS/MS spectra of m/z 598 (Peak 7). The structure of the 3,4-dihydrospirilloxanthin is shown, on this the fragmentation sites that would explain the m/z signals observed in the MS/MS spectrum are outlined. The presence of methoxy groups was observed through the loss of methanol (CH_3OH) or formaldehyde ($CH_2{=}O$), the elimination of methyl was not observed.

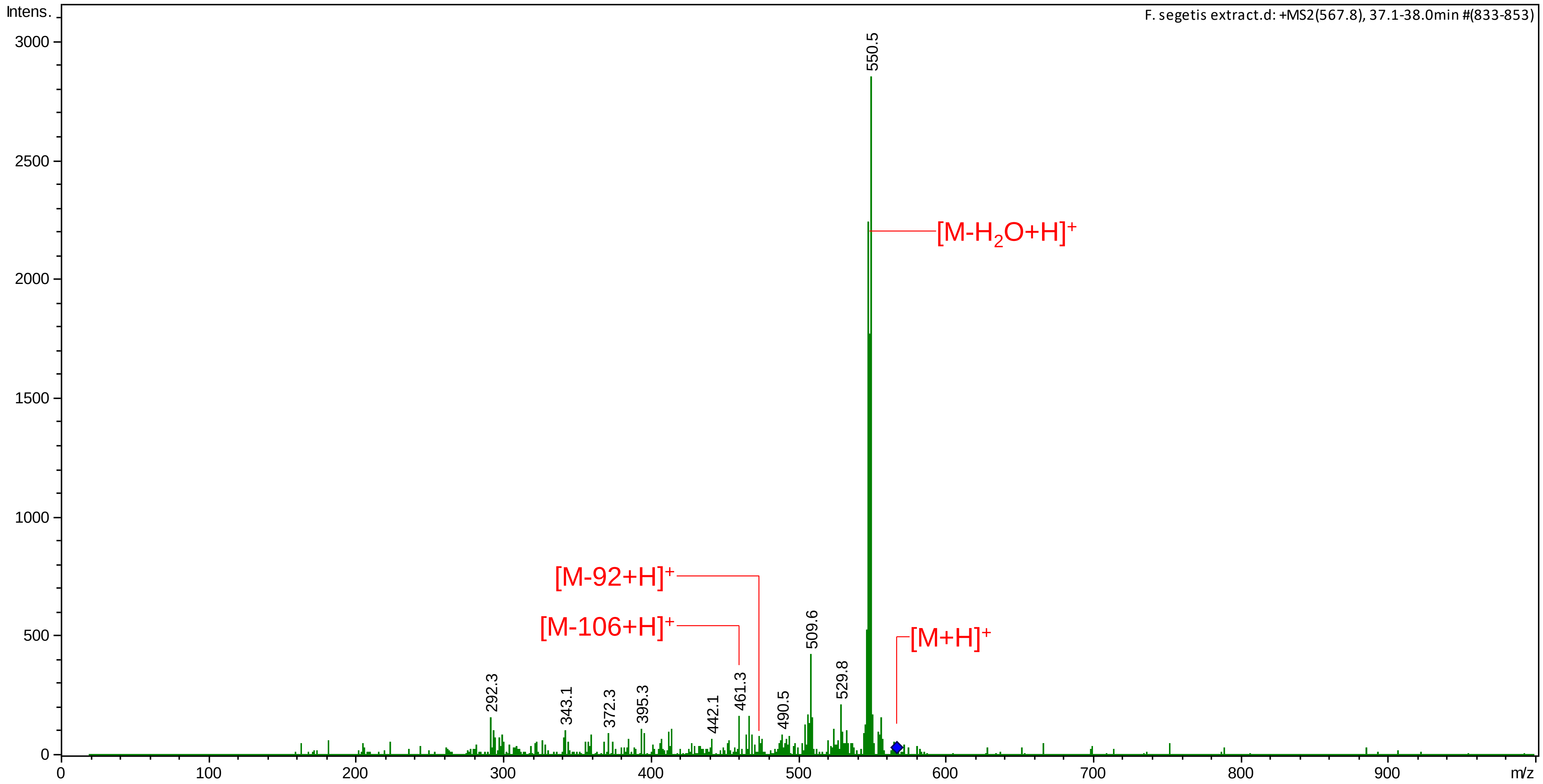

Figure S26. MS/MS spectra of m/z 567 (Peak 7). The assignment of some m/z signals are shown in red.

Hydroxyechinenone
$C_{40}H_{54}O_2$
MW 566 g/mol

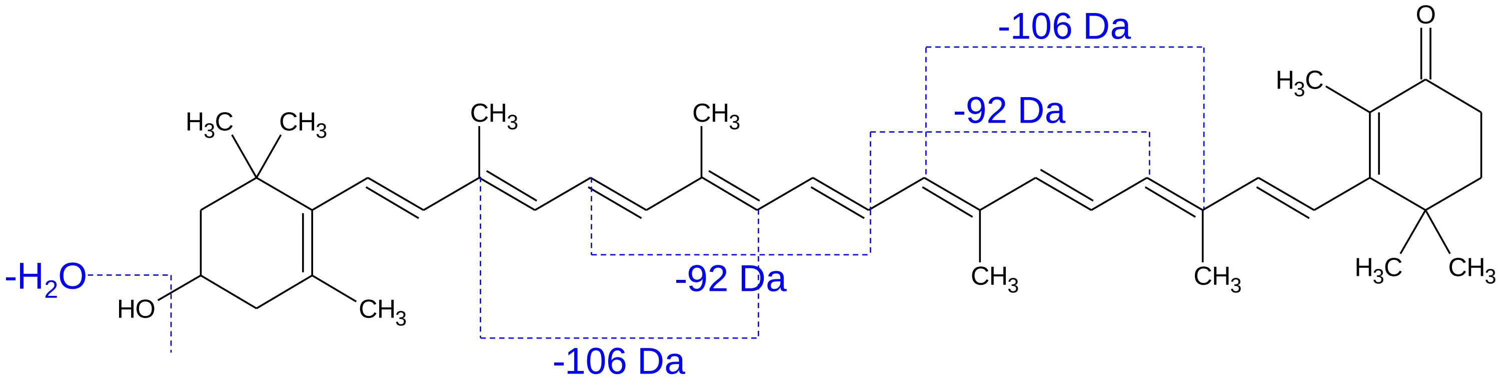

Figure S27. Interpretation of MS/MS spectra of m/z 567 (Peak 7). The structure of the hydroxyechinenone is shown, on this the fragmentation sites that would explain the m/z signals observed in the MS/MS spectrum are outlined.

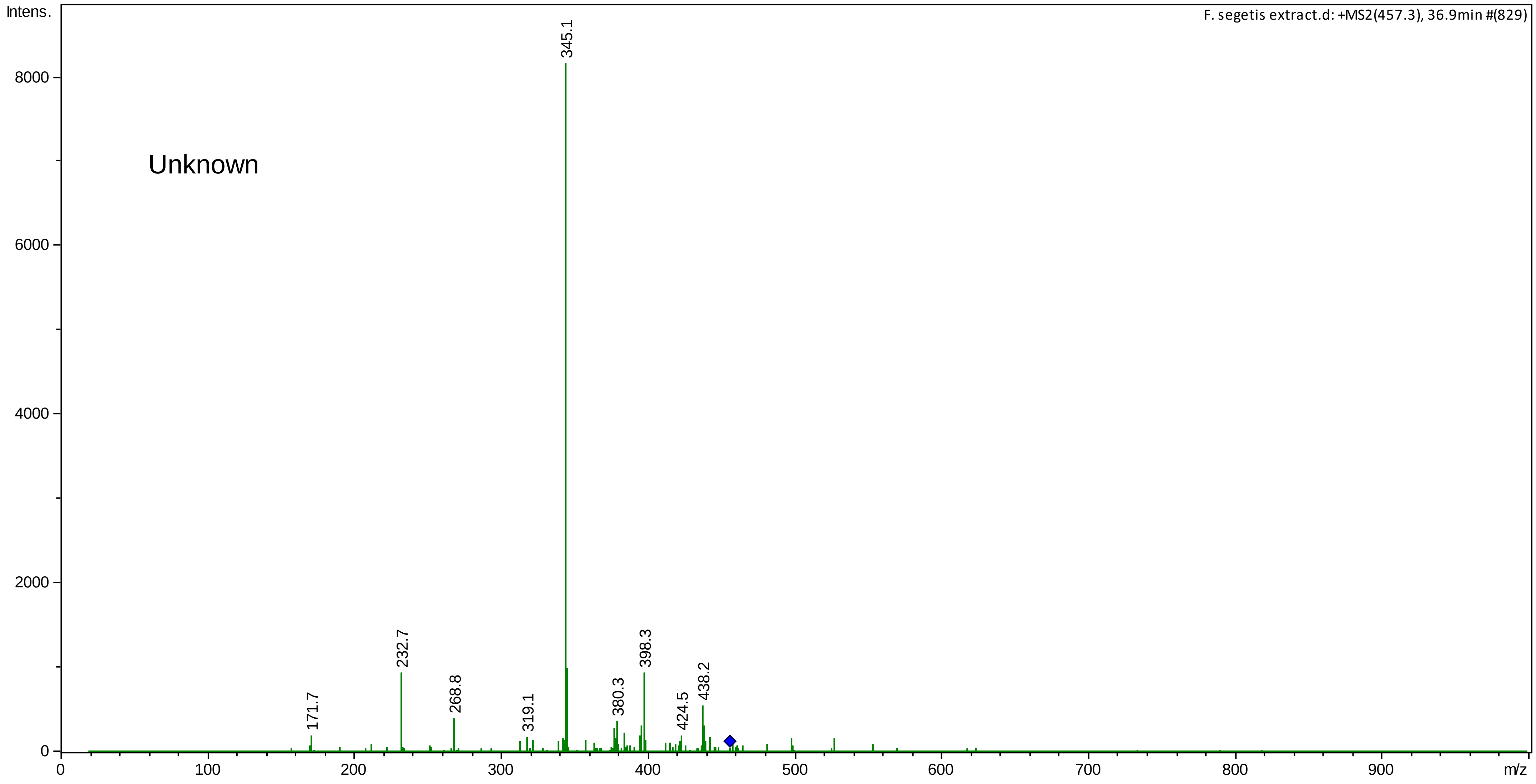

Figure S28. MS/MS spectra of m/z 457 (Peak 7).
